# Organometallic Half-Sandwich Dichloridoruthenium(II) Complexes with 7-Azaindoles: Synthesis, Characterization and Elucidation of Their Anticancer Inactivity against A2780 Cell Line

**DOI:** 10.1371/journal.pone.0143871

**Published:** 2015-11-25

**Authors:** Pavel Štarha, Lucie Hanousková, Zdeněk Trávníček

**Affiliations:** Regional Centre of Advanced Technologies and Materials, Department of Inorganic Chemistry, Faculty of Science, Palacký University in Olomouc, 17. listopadu 12, 771 46, Olomouc, Czech Republic; University of Edinburgh, UNITED KINGDOM

## Abstract

A series of organometallic half-sandwich dichloridoruthenium(II) complexes of the general formula [Ru(*η* ^6^-*p*-cym)(*n*aza)Cl_2_] (**1**–**8**; *p*-cym = *p*-cymene; *n*aza = 7-azaindole or its derivatives) was synthesised and fully characterized by elemental analysis, mass spectrometry, and infrared and multinuclear NMR spectroscopy. A single-crystal X-ray structural analysis of [Ru(*η* ^6^-*p*-cym)(*2Me4Cl*aza)Cl_2_] (**6**) revealed a typical piano-stool geometry with an *N*7-coordination mode of 2-methyl-4-chloro-7-azaindole (*2Me4Cl*aza). The complexes have been found to be inactive against human ovarian cancer cell line A2780 up to the highest applied concentration (IC_50_ > 50.0 μM). An inactivity of the complexes is caused by their instability in water-containing solvents connected with a release of the *n*aza *N*-donor ligand, as proved by the detailed ^1^H NMR, mass spectrometry and fluorescence experiments.

## Introduction

Platinum-based anticancer agents (e.g. *cisplatin*, *oxaliplatin* and *carboplatin*) are widely used for treating of various types of cancer [1–3]. However, their clinical utility is limited because many tumors are resistant to the platinum drugs and these cytostatics show several side effects including neurotoxicity, nephrotoxicity or myelosuppression. These drawbacks motivate bioinorganic chemists to development of complexes based on other, i.e. non-platinum, transition metals, which would offer wide range of anticancer efficacy and show reduced negative side effects. Among these compounds, the ruthenium-based antitumor agents represent a promising alternative to the clinically used platinum(II) compounds showing different mechanism of action connected with high cytotoxicity, different spectrum of efficiency against human cancer cell lines and the prospect of non cross-resistance [3–7]. Two ruthenium compounds, KP1019/NKP-1339 (indazolium or sodium salt of *trans*-[RuCl_4_(ind)_2_]; ind = indazole) and NAMI-A ((imH)[*trans*-RuCl_4_(DMSO)(im)]; im = imidazole), recently entered the clinical trials as prospective anticancer agents [8,9]. Many other ruthenium complexes were found to be highly *in vitro* and/or *in vivo* antitumor active against various types of tumors [10,11]. For example, complex [Ru(*η* ^6^-*p*-cym)(L_1_)Cl](PF_6_) was tested *in vitro* against ovarian A2780 (IC_50_ = 16.2 μM), lung A549 (IC_50_ = 10.5 μM), colon HCT116 (IC_50_ = 3.4 μM) and breast (IC_50_ = 12.1 μM) carcinoma human cancer cell lines; L_1_ = *N*,*N*-dimethyl-*N*-[(2-pyridinyl)methylene]-1,4-benzenediamine [10]. Another Ru complex, *trans*-[RuCl_4_(DMSO)(L_2_)]∙Me_2_CO, showed significantly higher (p < 0.05) *in vivo* antitumor activity against lymphocytic leukaemia L1210 (% T/C = 106) in comparison with aforementioned NAMI-A (% T/C = 94); L_2_ = 6-(2-bromobenzylamino)purine; % T/C is defined as the ratio of the mean survival time of the treated animal groups (T) divided by the mean survival of the untreated control group (C) [11].

**Fig 1:**
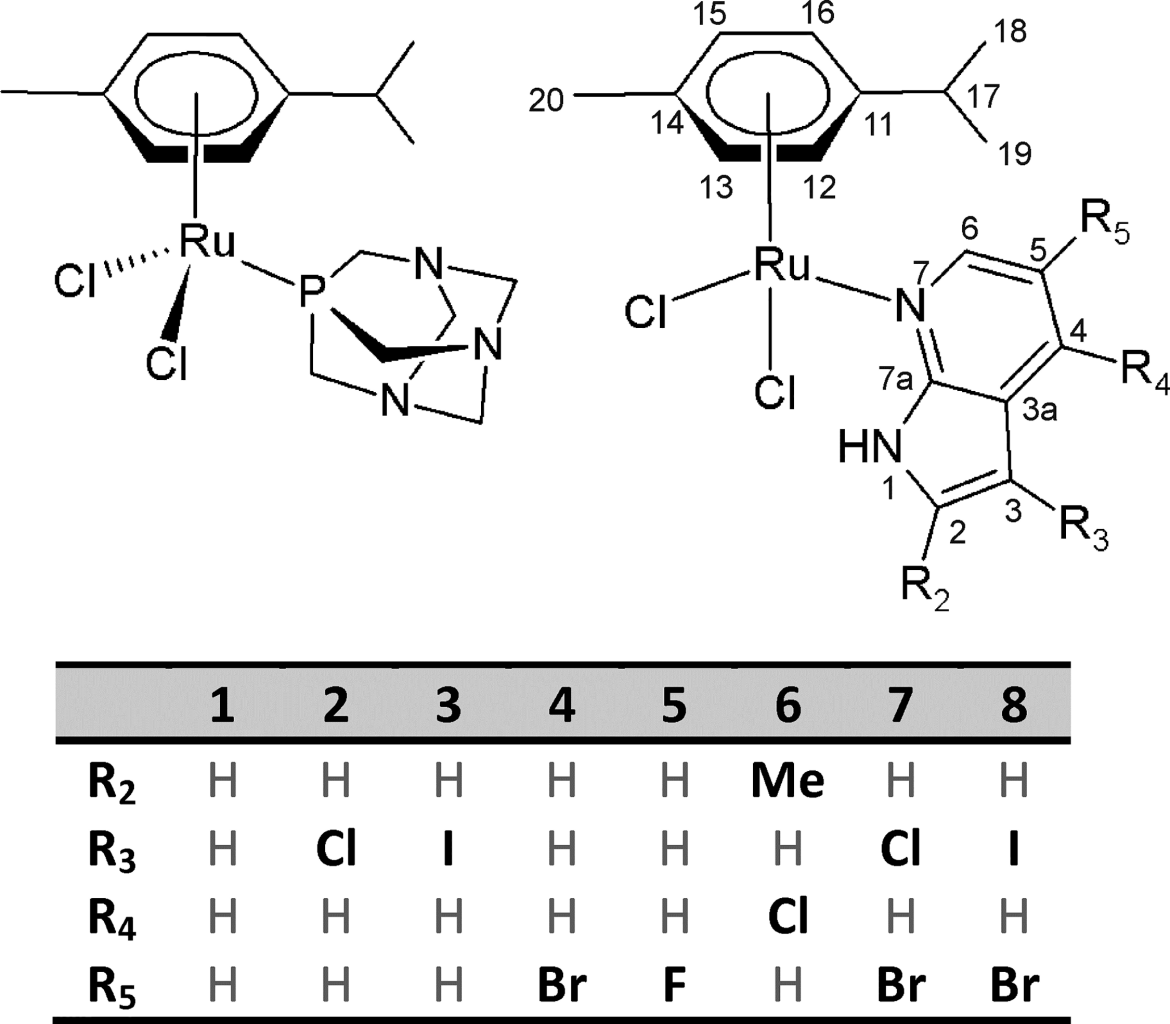
The structural formulas of RAPTA-C (*left*) and herein reported complexes 1–8 (*right*). The general structural formula of **1**–**8** is given together with the atom numbering scheme and with table providing the substituent specifications.

Half-sandwich organometallic ruthenium(II) complexes offer a broad scope for the design of therapeutic and diagnostic agents [10,12]. These complexes have attracted considerable interest as potential anticancer agents because of their efficacy against platinum drug-resistant tumors, selectivity and often good aqueous solubility. A representative of this group is a dichloridoruthenium(II) complex RAPTA-C, [Ru(*η* ^6^-*p*-cym)(pta)Cl_2_] (Fig 1; pta = 1,3,5-triaza-7-phosphatricyclo[3.3.1.1]decane), showing strong angiogenic effect [13]. Various RAPTA-C analogues of the general composition [Ru(*η* ^6^-*p*-cym)(L)Cl_2_] containing a monodentate-coordinated *N*-donor ligand (L) have been reported to date as being *in vitro* cytotoxic against various human cancer lines [14–22]. In particular, complex containing lonidamine-modified imidazole ligand exhibited IC_50_ values of 19.3, 17.9, 6.4, 8.3, 5.7, 20.5 and 20.2 μM against human *cisplatin*-sensitive (A2780) and -resistant (A2780R) ovarian cancer cell lines, human glioblastoma cell lines LN18, LN229, LNZ308, non-cancerous HEK cell line, and cortex neurons, respectively [14]. [Ru(*η* ^6^-*p*-cym)(L_3_)Cl_2_], where L_3_ = 4-[(10-hydroxydecyl)oxy]phenyl-4-pyridinecarboxylate, was found to be highly active against A2780 and A2780R (IC_50_ = 0.2 μM, and 3.0 μM, respectively) [15]. The complex [Ru(*η* ^6^-*p*-cym)(L_4_)Cl_2_] with 3-aza-5*H*-phenanthridin-6-one (L_4_) acting as monodentate *N*-donor ligand was moderately *in vitro* cytotoxic against human A549 lung, HCT116 colon, A2780 ovarian and Hcc1937 breast carcinoma cell lines, as well as against MRC5 normal lung fibroblast cell line with the IC_50_ values equalled 85.1, 38.8, 46.0, 93.3 and 143.0 μM, respectively [16]. *N*-[3-(imidazol-1-yl)propyl](4-dimethylamino)-1,8-naphthalimide (L_5_) was used as a monodentate *N*-donor ligand of highly active [Ru(*η* ^6^-*p*-cym)(L_5_)Cl_2_] complex having the IC_50_ values of 6.1, 7.8 and 12.7 μM against A2780, A2780R, and HEK, respectively [17]. The complex [Ru(*η* ^6^-*p*-cym)(NH_3_)Cl_2_] was inactive towards A2780 cells (IC_50_ > 100 μM) [18]. [Ru(*η* ^6^-*p*-cym)(L_6_)Cl_2_], where L_6_ stands for 5-fluorouracil-1-methylisonicotinate, was studied against promyelocytic leukaemia cells HL-60 (IC_50_ > 200 μM) and liver cancer cell line BEL-7420 (IC_50_ = 8.1 μM) [19]. [Ru(*η* ^6^-*p*-cym)(L_7_)Cl_2_] (L_7_ = 3-picoline) was tested towards the LoVo colon carcinoma (IC_50_ = 90 μM) and MiaPaCa pancreatic cancer (IC_50_ = 155 μM) cell lines [20]. The complex [Ru(*η* ^6^-*p*-cym)(L_8_)Cl_2_], which contains 4-(2-hydroxyethyl)pyridine (L_8_), was found to be low potent against HL-60 (IC_50_ = 202 μM) [21]. Finally, complex [Ru(*η* ^6^-*p*-cym)(L_9_)Cl_2_] containing mebendazole (L_9_) showed acute antitumor activity against HeLa cervical cancer cell line with IC_50_ = 0.20 μM [22].

Herein we report the synthesis and characterization of the complexes of the general composition [Ru(*η* ^6^-*p*-cym)(*n*aza)Cl_2_] (**1**–**8**; *p*-cym = *p*-cymene) containing 7-azaindole or its derivatives (*n*aza). The reported complexes **1**–**8** (Fig 1) were, due to known antitumor effect of their structural analogues containing different *N*-donor ligands, investigated for their *in vitro* cytotoxic effect against human ovarian cancer cell lines A2780. Because all the complexes were identified as cytotoxic inactive (IC_50_ ˃ 50.0 μM), we strived to investigate and explain the reasons of their inactivity by means of ^1^H NMR, ESI+ mass spectrometry and fluorescence studies.

## Materials and Methods

### Chemicals

The chemicals (RuCl_3_∙*x*H_2_O, 7-azaindole (aza), 3-iodo-7-azaindole (*3I*aza), 3-chloro-7-azaindole (*3Cl*aza), 5-bromo-7-azaindole (*5Br*aza), 5-fluoro-7-azaindole (*5F*aza), 2-methyl-4-chloro-7-azaindole (*2Me4Cl*aza), 3-chloro-5-bromo-7-azaindole (*3Cl5Br*aza), 3-iodo-5-bromo-7-azaindole (*3I5Br*aza), α-terpinene, *cisplatin* (CDDP), reduced glutathione (GSH), ethidium bromide (EtBr), tris(hydroxymethyl)aminomethane (TRIS)), solvents (methanol, diethyl ether, *n*-hexane, CDCl_3_, DMF-*d*_*7*_, MeOD-*d*_*4*_, D_2_O) and calf thymus DNA (ctDNA) were purchased from Sigma-Aldrich (Prague, Czech Republic), Acros Organics (Pardubice, Czech Republic) and Precious Metals Online (University of Wollongong, Australia). [Ru(μ-Cl)(*η* ^6^-*p*-cym)Cl]_2_ was prepared according to the reported synthetic procedure [23].

### Synthesis

[Ru(μ-Cl)(*η* ^6^-*p*-cym)Cl]_2_ (0.1 mmol, 61.2 mg) was dissolved in methanol (5 mL) and left to react with two molar equivalents of the corresponding *n*aza. The reaction mixture was stirred at ambient temperature for 20 min (reactions with *5Br*aza and *5F*aza), 2 h (reactions with aza, *3I*aza and *3Cl*aza) or 3 h (reactions with *2Me4Cl*aza, *3Cl5Br*aza and *3I5Br*aza), until the yellow (for **2**, **4** and **5**), or light (for **8**) or dark (for **1**, **3**, **6** and **7**) orange product was formed. The products (Fig 1) were isolated by filtration, washed with methanol (2 × 2 mL) and diethyl ether (3 × 5 mL) and dried under the infrared lamp (40°C, 4 h). The yields were 60–80%.

[Ru(*η* ^6^-*p*-cym)(aza)Cl_2_] (**1**): *Anal*. Calcd. for RuCl_2_C_17_H_20_N_2_: C, 48.12; H, 4.75; N, 6.60; found: C, 47.76; H, 4.74; N, 6.37%. ^1^H NMR (CDCl_3_, ppm): δ 11.00 (s, N1–H, 1H), 8.68 (d, *J* = 5.5, C6–H, 1H), 7.94 (d, *J* = 7.8, C4–H, 1H), 7.31 (d, *J* = 2.8, C2–H, 1H), 7.11 (dd, *J* = 7.9, C5–H, 1H), 6.44 (dd, *J* = 3.4, C3–H, 1H), 5.58 (d, *J* = 6.2, C13–H, C15–H, 2H), 5.28 (d, *J* = 5.5, C12–H, C16–H, 2H), 2.96 (sep, *J* = 6.9, C17–H, 1H), 1.83 (s, C20–H, 3H), 1.19 (d, *J* = 6.7, C18–H,C19–H, 6H). ^13^C NMR (CDCl_3_, ppm): δ 151.1 (C6), 148.7 (C7a), 130.7 (C4), 126.3 (C2), 122.9 (C3a), 166.7 (C5), 103.4 (C14), 101.9 (C3), 97.7 (C11), 83.6 (C12, C16), 81.7 (C13, C15), 30.6 (C17), 22.3 (C18, C19), 18.1 (C20).

[Ru(*η* ^6^-*p*-cym)(*3Cl*aza)Cl_2_] (**2**): *Anal*. Calcd. for RuCl_3_C_17_H_19_N_2_: C, 44.51; H, 4.18; N, 6.11; found: C, 44.32; H, 4.19; N, 5.95%. ^1^H NMR (CDCl_3_, ppm): δ 11.12 (s, N1–H, 1H), 8.78 (d, *J* = 5.1, C6–H, 1H), 7.99 (d, *J* = 7.8, C4–H, 1H), 7.29 (s, C2–H, 1H), 7.21 (t, *J* = 6.3, C5–H, 1H), 5.60 (d, *J* = 5.5, C13–H, C15–H, 2H), 5.31 (d, *J* = 5.5, C12–H, C16–H, 2H), 2.95 (sep, *J* = 7.0, C17–H, 1H), 1.85 (s, C20–H, 3H), 1.30 (d, *J* = 7.0, C18–H, C19–H, 6H). ^13^C NMR (CDCl_3_, ppm): δ 150.0 (C6), 149.4 (C7a), 128.9 (C4), 123.1 (C2), 120.8 (C3a), 117.1 (C5), 103.4 (C14), 105.6 (C3), 97.8 (C11), 81.6 (C12, C16), 83.7 (C13, C15), 30.6 (C17), 22.3 (C18, C19), 18.1 (C20).

[Ru(*η* ^6^-*p*-cym)(*3I*aza)Cl_2_] (**3**): *Anal*. Calcd. for RuCl_2_C_17_H_19_N_2_I: C, 37.11; H, 3.48; N, 5.09; found: C, 37.05; H, 3.34; N, 5.47%. ^1^H NMR (CDCl_3_, ppm): δ 11.29 (s, N1–H, 1H), 8.76 (d, *J* = 4.8, C6–H, 1H), 7.79 (d, *J* = 7.6, C4–H, 1H), 7.37 (s, C2–H, 1H), 7.21 (t, *J* = 7.6, 5.5, C5–H, 1H), 5.61 (d, *J* = 5.5, C13–H, C15–H, 2H), 5.32 (d, *J* = 5.5,C12–H, C16–H, 2H), 2.95 (sep, *J* = 6.8, C17–H, 1H), 1.84 (s, C20–H, 3H), 1.30 (d, *J* = 6.9, C18–H,C19–H, 6H). ^13^C NMR (CDCl_3_, ppm): δ 150.5 (C6), 150.0 (C7a), 131.5 (C4), 131.0 (C2), 125.3 (C3a), 117.6 (C5), 103.5 (C14), 98.1 (C11), 83.9 (C13, C15), 81.8 (C12, C16), 30.8 (C17), 22.5 (C18, C19), 18.3 (C20).

[Ru(*η* ^6^-*p*-cym)(*5Br*aza)Cl_2_] (**4**): *Anal*. Calcd. for RuCl_2_C_17_H_19_N_2_Br: C, 40.57; H, 3.81; N, 5.57; found: C, 40.80; H, 3.82; N, 5.47%. ^1^H NMR (CDCl_3_, ppm): δ 11.19 (s, N1–H, 1H), 8.77 (d, *J* = 2.1, C6–H, 1H), 8.09 (d, *J* = 1.4, C4–H, 1H), 7.32 (d, *J* = 2.9, C2–H, 1H), 6.47 (dd, *J* = 3.4, C3–H, 1H), 5.60 (d, *J* = 6.2, C13–H, C15–H, 2H), 5.31 (d, *J* = 6.2, C12–H, C16–H, 2H), 2.95 (sep, *J* = 6.9, C17–H, 1H), 1.87 (s, C20–H, 3H), 1.31 (d, *J* = 6.9, C18–H,C19–H, 6H). ^13^C NMR (CDCl_3_, ppm): δ 148.9 (C7a), 133.0 (C4), 127.9 (C2), 123.9 (C3a), 111.2 (C5), 103.4 (C14), 101.5 (C3), 97.8 (C11), 83.7 (C12, C16), 81.7 (C13, C15), 29.7 (C17), 22.3 (C18, C19), 18.1 (C20).

[Ru(*η* ^6^-*p*-cym)(*5F*aza)Cl_2_] (**5**): *Anal*. Calcd. for RuCl_2_C_17_H_19_N_2_F: C, 46.16; H, 4.33; N, 6.33; found: C, 45.90; H, 4.42; N, 6.10%. ^1^H NMR (CDCl_3_, ppm): δ 11.08 (s, N1–H, 1H), 8.67 (dd, *J* = 3.8, 2.4, C6–H, 1H), 7.70 (dd, *J* = 7.9, 2.4, C4–H, 1H), 7.39 (t, *J* = 2.8, C2–H, 1H), 6.50 (dd, *J* = 3.4, 2.1, C3–H, 1H), 5.60 (d, *J* = 6.2, C13–H, C15–H, 2H), 5.31 (d, *J* = 6.2, C12–H, C16–H, 2H), 2.97 (sep, *J* = 6.9, C17–H, 1H), 1.87 (s, C20–H, 3H), 1.31 (d, *J* = 7.0, C18–H, C19–H, 6H). ^13^C NMR (CDCl_3_, ppm): δ 148.6 (C7a), 131.1 (C4), 137.6 (C6), 128.9 (C2), 122.7 (C3a), 117.0 (C5), 103.6 (C14), 102.3 (C3), 98.0 (C11), 83.9 (C12, C16), 81.1 (C13, C15), 30.8 (C17), 22.5 (C18, C19), 18.3 (C20).

[Ru(*η* ^6^-*p*-cym)(*2Me4Cl*aza)Cl_2_] (**6**): *Anal*. Calcd. for RuCl_3_C_18_H_21_N_2_: C, 45.73; H, 4.48; N, 5.92; found: C, 45.40; H, 4.49; N, 5.60%. ^1^H NMR (CDCl_3_, ppm): δ 11.00 (s, N1–H, 1H), 8.45 (d, *J* = 5.9, C6–H, 1H), 7.07 (d, *J* = 6.3, C5–H, 1H), 6.26 (s, C3–H, 1H), 5.54 (d, *J* = 5.5, C13–H, C15–H, 2H), 5.27 (d, *J* = 5.5, C12–H, C16–H, 2H), 2.96 (sep, *J* = 6.5, C17–H, 1H), 2,47 (s, C10–H, 3H), 1.90 (s, C20–H, 3H), 1.30 (d, *J* = 6.7, C18–H, C19–H, 6H). ^13^C NMR (CDCl_3_, ppm): δ 151.3 (C6), 147.2 (C7a), 138.4 (C2), 122.9 (C3a), 119.9 (C5), 98.2 (C3), 103.5 (C14), 97.5 (C11), 83.2 (C12, C16), 81.8 (C13, C15), 30.5 (C18), 22.2 (C19, C20), 18.1 (C17), 14.2 (C8).

[Ru(*η* ^6^-*p*-cym)(*3Cl5Br*aza)Cl_2_] (**7**): *Anal*. Calcd. for RuCl_3_C_17_H_18_N_2_Br: C, 37.98; H, 3.37; N, 5.21; found: C, 37.88; H, 3.27; N, 4.99%. ^1^H NMR (CDCl_3_, ppm): δ 11.25 (s, N1–H, 1H), 8.84 (s, C6–H, 1H), 8.12 (s, C4–H, 1H), 7.32 (s, C2–H, 1H), 5.60 (d, *J* = 5.9, C13–H, C15–H, 2H), 5.32 (d, *J* = 5.9, C12–H, C16–H, 2H), 2.93 (sep, *J* = 5.9, C17–H, 1H), 1.89 (s, C20–H, 3H), 1.31 (d, *J* = 7.3, C18–H, C19–H, 6H). ^13^C NMR (CDCl_3_, ppm): δ 151.2 (C6), 148.3 (C7a), 131.4 (C4), 128.8 (C2), 121.8 (C3a), 111.9 (C5), 105.2 (C3), 103.6 (C14), 98.0 (C11), 83.8 (C12, C16), 81.8 (C13, C15), 30.8 (C17), 22.4 (C18, C19), 18.3 (C20).

[Ru(*η* ^6^-*p*-cym)(*3I5Br*aza)Cl_2_] (**8**): *Anal*. Calcd. for RuCl_2_C_17_H_18_N_2_BrI: C, 32.46; H, 2.88; N, 4.45; found: C, 32.24; H, 2.77; N, 4.22%. ^1^H NMR (CDCl_3_, ppm): δ 11.38 (s, N1–H, 1H), 8.79 (s, C6–H, 1H), 7.91 (d, *J* = 1.4, C4–H, 1H), 7.39 (d, *J* = 2.7, C2–H, 1H), 5.58 (d, *J* = 5.5, C13–H, C15–H, 2H), 5.30 (d, *J* = 5.5, C12–H, C16–H, 2H), 2.91 (sep, *J* = 6.9, C17–H, 1H), 1.86 (s, C20–H, 3H), 1.29 (d, *J* = 6.9, C18–H, C19–H, 6H). ^13^C NMR (CDCl_3_, ppm): δ 149.9 (C6), 149.3 (C7a), 133.8 (C4), 132.2 (C2), 126.1 (C3a), 112.1 (C5), 103.4 (C14), 97.9 (C11), 83.7 (C13, C15), 81.6 (C12, C16), 30.6 (C17), 22.2 (C18, C19), 18.2 (C20).

### General methods

^1^H, ^13^C, ^1^H–^1^H gs-COSY, ^1^H–^13^C gs-HMQC and ^1^H–^13^C gs-HMBC spectra were acquired at 298 K on a JEOL JNM-ECA 600II device at 600.00 MHz (^1^H) and 150.86 MHz (^13^C); gs = gradient selected, COSY = correlation spectroscopy, HMQC = heteronuclear multiple quantum coherence, HMBC = heteronuclear multiple bond coherence). The splitting of proton resonances in the reported ^1^H spectra is defined as s = singlet, d = doublet, dd = doublet of doublets, t = triplet, sep = septet. Spectra were calibrated using protio impurity signals of used solvent—for CDCl_3_: 7.26 ppm (^1^H NMR) and 77.16 ppm (^13^C NMR); for MeOD-*d*_*4*_: 3.31 ppm (^1^H NMR); for DMF-*d*_*7*_: 8.03, 2.92 and 2.75 ppm (^1^H NMR); for 10% DMF-*d*_*7*_/D_2_O and 10% MeOD-*d*_*4*_/D_2_O: 4.79 ppm (for D_2_O in ^1^H NMR). Electrospray ionization (ESI) mass spectra of the methanol solution were obtained of on a LCQ Fleet Ion Trap mass spectrometer (Thermo Scientific; Qual Browser software, version 2.0.7) in the positive ionization mode (ESI+). Elemental analysis (C, H, N) was performed using a Flash 2000 CHNS Elemental Analyzer (Thermo Scientific). FTIR spectra were obtained on a Nexus 670 FT-IR (Thermo Nicolet) on an ATR diamond plate between 400 and 4000 cm^–1^.

### Single-crystal X-ray analysis of [Ru(*η* ^6^-*p*-cym)(*2Me4Cl*aza)Cl_2_] (6)

Single crystals of [Ru(*η* ^6^-*p*-cym)(*2Me4Cl*aza)Cl_2_] (**6**) were grown from its saturated MeOH solution by slow evaporation method after 48 h of standing at ambient temperature. X-ray diffraction data of **6** were collected with a Rigaku HighFlux HomeLab™ universal dual wavelength (Mo–Kα and Cu–Kα) single crystal diffractometer at 120(2) K, using the Mo-Kα radiation (λ = 0.71075 Å). The diffractometer was equipped with the Eulerian 3 circle goniometer and the Rigaku Saturn724+ (2 × 2 bin mode) detector. Data reduction and correction of the absorption effect were performed using the XDS software package [24]. The structure was solved by direct methods using SHELXS and refined on *F* ^*2*^ using the full-matrix least-squares procedure (SHELXL) [25]. Non-hydrogen atoms were refined anisotropically and all H-atoms were found from difference Fourier maps and refined using a riding model, with the AFIX 14, AFIX 43 and AFIX 137 instructions.

X-ray crystallographic data for **6** have been deposited in the Cambridge Structural Database under the accession Cambridge Crystallographic Data Centre number CCDC 1416250. The crystal data and structure refinements are given in S1 Table. The molecular graphics were drawn and additional structural calculations were interpreted using DIAMOND [26] and Mercury [27].

### Cell culture and *in vitro* cytotoxicity testing

The *in vitro* cytotoxicity towards human ovarian carcinoma A2780 (ECACC No. 93112519) was tested by an MTT assay evaluated spectrophotometrically at 540 nm (TECAN, Schoeller Instruments LLC). The cancer cell lines were cultured according to the ECACC instructions and were maintained at 37°C and 5% CO_2_ in humidified incubator. **1**–**8**, *cisplatin* and *n*aza (0.01–50.0 μM concentrations) interacted with the cancer cells for 24 h, using 96-well culture plates. The cells was tested in parallel with vehicle (DMF; 0.1%, v/v), and Triton X-100 (1%, v/v) to assess the minimal (100% viability) and maximal (0% viability) cell damage, respectively. The cytotoxicity data were received from three independent experiments (each conducted in triplicate) using cells from three consecutive passages.

### Studies of solvolysis and interactions with glutathione (GSH)

The representative complexes **2** and **8** (1 mM in DMF-*d*_*7*_, MeOD-*d*_*4*_, 10% DMF-*d*_*7*_/D_2_O and 10% MeOD-*d*_*4*_/D_2_O) or their mixtures with 2 molar equivalents of GSH in 10% MeOD-*d*_*4*_/D_2_O were studied by ^1^H NMR (acquired at 298 K on JEOL JNM-ECA 600II) right after the preparation of the studied solutions (0 h) and after 1 h, 24 h and 48 h of standing at ambient temperature. Analogical experiments were performed in non-deuterated solvents (10% DMF-*d*_*7*_/D_2_O, 10% MeOH/H_2_O) and analysed by ESI+ mass spectrometry (LCQ Fleet Ion Trap mass spectrometer).

### Fluorescence quenching experiments

ctDNA (154 μM) and EtBr (3 mM) were mixed together in TRIS/NaCl buffer (pH = 7.2) and incubated for 30 min at ambient temperature. 0, 100, 200, 400, 600 and 1000 μL of the representative complexes **2**, **5** and **8** (stock solutions of the 150 μM concentration) dissolved in 10% methanol solution in TRIS/NaCl buffer were added to the EtBr/ctDNA system and the volume was refilled to 3 mL with TRIS/NaCl buffer. The mixtures were incubated at ambient temperature for next 15 min. The emitted fluorescence (546 nm excitation wavelength) was recorded on a fluorescence spectrometer AvaSpec HS1024x122TE using a 1 cm quartz cell.

## Results and Discussion

This work aimed to prepare and study the *in vitro* cytotoxicity and basic mechanistic aspects of half-sandwich organometallic dichloridoruthenium(II) complexes of the general formula [Ru(*η* ^6^-*p*-cym)(*n*aza)Cl_2_] containing 7-azaindole or its derivatives (*n*aza) as monodentate *N*-donor ligands. Many representatives of this type of ruthenium(II) organometallic complexes have been recently reported by several research groups as antitumor active against various human cancer cell lines (see Introduction) [14–22]. In the case of this work, the 7-azaindole or its derivatives were used, because these simple organic compounds were recently shown as suitable monodentate *N-*donor ligands of biologically active transition metal complexes, such as *cis*-dichloridoplatinum(II) complexes (e.g. references [28–30]) showing up to ca 14-fold cytotoxic effect against various human cancer cell lines. For example, *cis*-[PtCl_2_(*5Br*aza)_2_] exhibited IC_50_ = 1.8, 2.0 and 0.6 μM against A2780, HOS osteosarcoma, and G-361 malignant melanoma, respectively (IC_50_ = 12.0, 34.2, and 3.4 μM, respectively, for CDDP) [28–29].

### Chemistry

#### Synthesis and general properties

**Fig 2:**
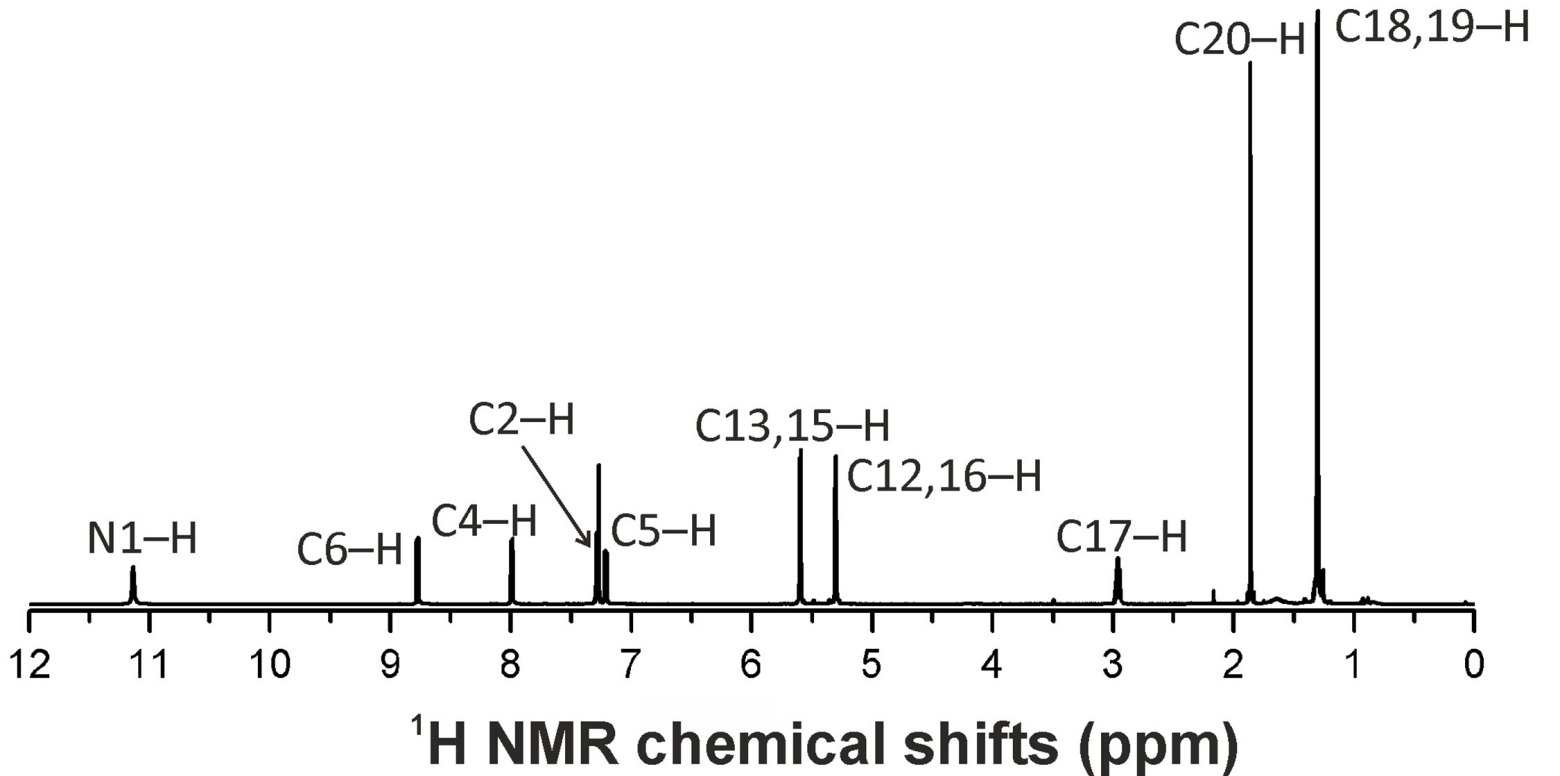
^1^H NMR spectrum of 2 in CDCl_3_ given together with assignment of the hydrogen atoms.

A series of eight complexes of the general formula [Ru(*η* ^6^-*p*-cym)(*n*aza)Cl_2_] (**1**–**8**; Fig 1) was prepared by a one-step synthesis using the reaction of the stoichiometric amounts of the [Ru(μ-Cl)(*η* ^6^-*p*-cym)Cl]_2_ dimer and appropriate *n*aza, as described for analogical [Ru(*η* ^6^-*p*-cym)(L)Cl_2_] [14–22]. The reaction time differed depending on the substituent position on the 7-azaindole moiety. In particular, **4** and **5** containing 5-monosubstituted 7-azaindoles formed almost immediately and were isolable after 20 min of stirring at ambient temperature. For the complexes **1**–**3** and **6**–**8**, the reaction time was longer (2 or 3 h), because product formed gradually during this period of time. The complexes **1**–**8** were obtained in the yields of ca 60–80% (related to the starting Ru(II) dimer) and their composition and chemical purity were proved by a combination of elemental analysis, ^1^H NMR spectroscopy in CDCl_3_ (Fig 2) and a single-crystal X-ray structural analysis.

All the signals of the coordinated *p*-cymene and 7-azaindole-based ligands were found in ^1^H (with the appropriate integral intensities) and ^13^C NMR spectra recorded on CDCl_3_ solutions of **1**–**8** (see Synthesis section). As an example, Fig 2 shows the ^1^H NMR spectrum of **2** in CDCl_3_. The spectra did not change in time (recorded after 24 and 48 h), which indicated that **1**–**8** are stable in chloroform.

The ESI+ mass spectra were recorded on MeOH solutions of **1**–**8**. The peaks of the monochlorido [Ru(*η* ^6^-*p*-cym)(*n*aza)Cl]^+^ species were found for **1**–**6** and **8** (see S1 Fig for **8**), in the case of **3** the [Ru(*η* ^6^-*p*-cym)(*3I*aza)Cl]^+^ peak was detected as overlapped with [Ru(*η* ^6^-*p*-cym)(*3I*aza)(OCH_3_)]^+^ in the 2:1 ratio. Moreover, the peaks of the {[Ru(*η* ^6^-*p*-cym)(*n*aza)]–H}^+^ species were found in the ESI+ mass spectra of **1**, **2**, **6** and **7**. Further, the intensive peaks at 271.0, 507.0, 542.9, 570.9 and 576.9 *m/z* were observed in the ESI+ mass spectra of **1**–**8**, whose mass and isotopic distribution corresponded to {[Ru(*η* ^6^-*p*-cym)Cl]}^+^, [Ru_2_(*η* ^6^-*p*-cym)_2_Cl]^+^, {[Ru_2_(*η* ^6^-*p*-cym)_2_Cl_2_]+H}^+^, [Ru_2_(*η* ^6^-*p*-cym)_2_Cl_2_(OCH_3_)]^+^ and [Ru_2_(*η* ^6^-*p*-cym)_2_Cl_3_]^+^), i.e. the peaks detected also in the spectra of the starting ruthenium(II) dimer (S2 Fig). Finally, the ESI+ mass spectra of **1**–**8** also contained the peaks of {(*n*aza)+H}^+^ corresponding to the free *n*aza species.

#### Molecular and crystal structure of 6

**Fig 3:**
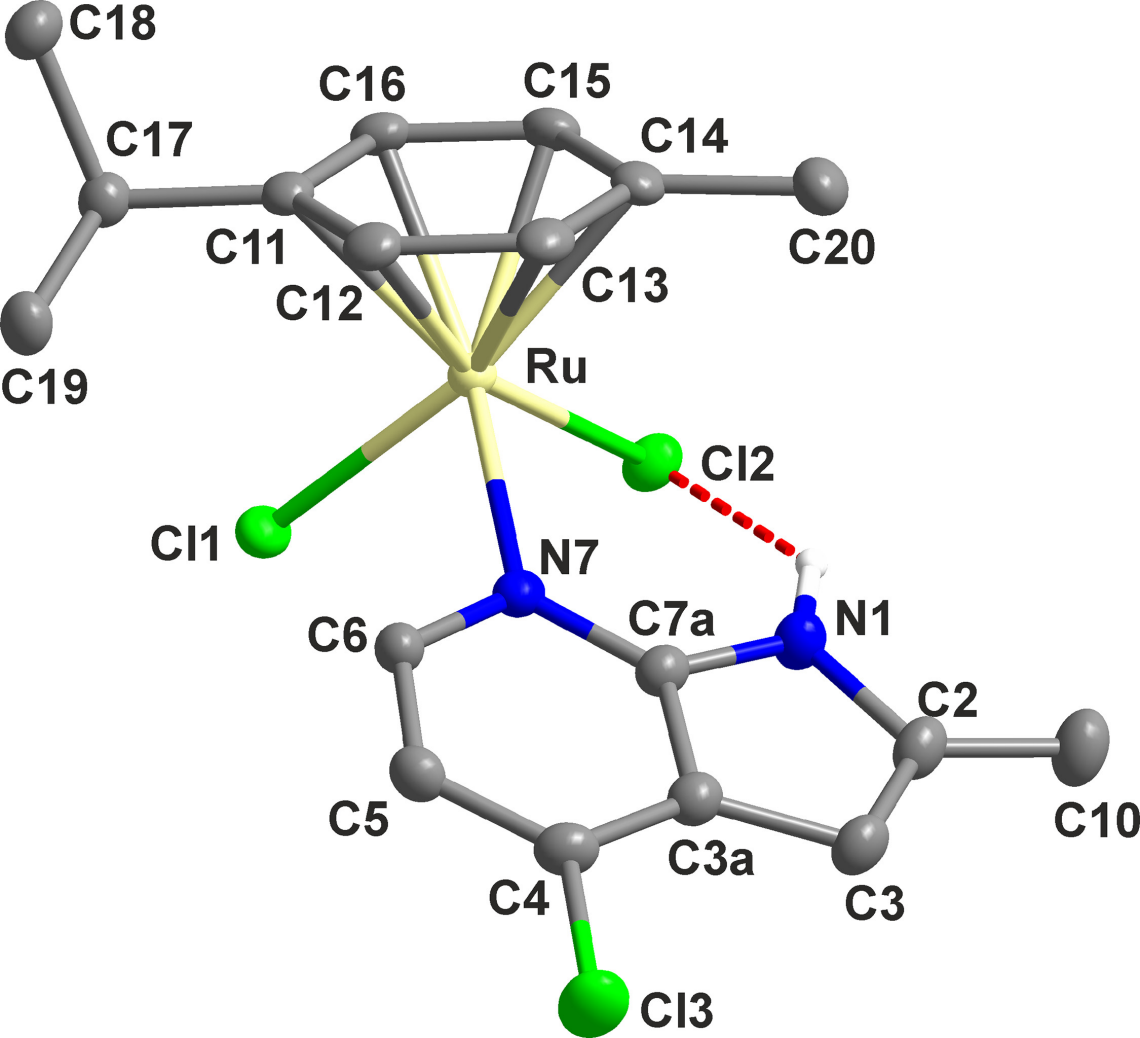
The molecular structure of [Ru(*η* ^6^-*p*-cym)(*2Me4Cl*aza)Cl_2_] (6). Non-hydrogen atoms are drawn as thermal ellipsoids at the 50% probability level and hydrogen atoms, except for the N1–H hydrogen involved in the depicted intramolecular N1–H···Cl2 hydrogen bond (red dashed line), were omitted for clarity. *p*-cym = *p*-cymene, *2Me4Cl*aza = 2-methyl-4-chloro-7-azaindole.

The crystals of [Ru(*η* ^6^-*p*-cym)(*2Me4Cl*aza)Cl_2_] (**6**) suitable for a single-crystal X-ray analysis were prepared by a diffusion of diethyl ether into the saturated chloroform solution of **6**. The molecular and crystal structures are depicted in Fig 3, and S3 Fig, respectively, while the selected bond lengths and angles can be found in Table 1.

**Table 1:** Selected bond lengths (Å) and angles (°) of [Ru(*η* ^6^-*p*-cym)(*2Me4Cl*aza)Cl_2_] (6).

Bond lengths (Å)		Bond angles (°)	
Ru1−Cl1	2.4163(5)	Cl1–Ru1–Cl2	86.82(2)
Ru1−Cl2	2.4254(4)	Cl1–Ru1–N7	85.23(4)
Ru1−N7	2.160(2)	Cl1–Ru1–Cg^a^	129.141(2)
Ru1−C11	2.208(2)	Cl2–Ru1–N7	91.50(4)
Ru1−C12	2.182(2)	Cl2–Ru1–Cg^a^	125.416(3)
Ru1−C13	2.163(2)	N7–Ru1–Cg^a^	125.82(4)
Ru1−C14	2.196(2)	Ru1–N7–C6	117.46(12)
Ru1−C15	2.174(2)	Ru1–N7–C7a	126.93(12)
Ru1−C16	2.189(2)		
Ru1–Cg^a^	1.660		

^a^) Cg = the centroid of the *p*-cymene aromatic ring

The complex **6** adopts the pseudo-octahedral piano-stool geometry (Fig 3) known to be typical for the structural analogues. To date, forty four half-sandwich *p*-cymene-dichloridoruthenium(II) complexes containing various heterocyclic ligands coordinated through endocyclic sp^2^ nitrogen heteroatom have been deposited within the Cambridge Structural Database (CSD ver. 5.36, May 2015 update [31]), as it can be exemplified on [Ru(*η* ^6^-*p*-cym)(L)Cl_2_], where L = *N*-(aminopropyl)imidazole derivative of lonidamine [14], 4-ethoxyphenyl-isonicotinate [15], nicotinamide [16], methyl isonicotinate [19], 4-picoline, 3,4-dimethylpyridine or *p*-toluidine [20] or 4-(2-hydroxyethyl)pyridine [21].

The Ru(II) atom of **6** is *η* ^6^-π-bonded to the arene ring of *p*-cymene, and coordinated by two chlorido ligands and one *2Me4Cl*aza molecule monodentate-coordinated to the metal centre through the pyridyl nitrogen (*N*7). An *N*7-coordination mode of *2Me4Cl*aza is consistent with the formerly reported ruthenium(II) complexes [Ru_2_(μ-L_10_)_4_(aza)_2_] [32] and [Ru_2_(μ-ac)_4_(aza)_2_]PF_6_∙CH_2_Cl_2_ [33] containing a monodentate-coordinated unsubstituted 7-azaindole (aza); L_10_ = 2-fluorobenzoate anion; ac = acetate anion. The Ru–N bond length of **6** (Table 1) is markedly lower as compared with aforementioned ruthenium(II) complexes containing aza, ranging from 2.288 to 2.364 Å. On the other hand, the Ru–N bond length of **6** fell into the 2.097–2.215 Å interval (average of 2.136 Å) found for the mentioned forty four structural analogues of the general composition [Ru(*η* ^6^-*p*-cym)(L)Cl_2_] deposited to date within CSD. Similarly, the Ru–Cl bond lengths of **6** (Table 1) fell into the 2.393–2.445 Å range discovered for formerly deposited [Ru(*η* ^6^-*p*-cym)(L)Cl_2_] complexes. The Ru···Cg distance between the metal centre and centroid of *p*-cymene aromatic ring (Table 1) correlates with the literature data reported for analogical complexes, such as [Ru(*η* ^6^-*p*-cym)(NH_3_)Cl_2_] with 1.657 Å [18] or [Ru(*η* ^6^-*p*-cym)(L_11_)Cl_2_] with 1.663 Å (L_11_ = methyl isonicotinate) [19]. The dihedral angle formed by the aromatic ring of *p*-cymene and 7-azaindole moiety is 29.98(4°.

The crystal structure involves intramolecular N1–H···Cl2 hydrogen bond with *d*(N1···Cl2) = 3.030(2) Å and <(N1–H···Cl2) = 135.36(12° (Fig 3), and several intermolecular non-covalent contacts of the C–H···Cl, C–H···C and C···Cl type (S2 Table, S3 Fig). Interestingly, no intermolecular π-π stacking interactions were found in the crystal structure of **6**.

### Studies of *in vitro* cytotoxicity and related mechanistic aspects

#### *In vitro* cytotoxicity against the A2780 cell line

All the prepared complexes were studied for their *in vitro* cytotoxicity against A2780 ovarian carcinoma cells, known to be sensitive for analogical organometallic dichloridoruthenium(II) complexes [14–17]. For example, structurally similar [Ru(*η* ^6^-*p*-cym)(L)Cl_2_] complexes containing lonidamine-modified imidazole ligand (IC_50_ = 19.3 μM) [14], 4-[(10-hydroxydecyl)oxy]phenyl-4-pyridinecarboxylate (IC_50_ = 0.2 μM) [15], 3-aza-5*H*-phenanthridin-6-one (IC_50_ = 38.8 μM) [16] or *N*-[3-(imidazol-1-yl)propyl](4-dimethylamino)-1,8-naphthalimide (IC_50_ = 6.1 μM) [17] were recently reported as highly active against the A2780 cell line. However, all the studied complexes **1**–**8** were found to be inactive up to the highest tested concentration (IC_50_ > 50.0 μM).

With respect to the aforementioned findings regarding the mutually different *in vitro* cytotoxicity of **1**–**8** against A2780, as compared with their analogues containing different monodentate *N*-donor ligands [14–17], we decided to perform several relevant experiments (^1^H NMR, ESI+ mass spectrometry), designed to shed a light on the reasons of inactivity of **1**–**8** (studied for the representative complexes **2** and **8**). The experiments were designed to prove whether: 1/ the complexes are stable under the used experimental condition and inactivity is due to low sensitivity of the A2780 cells towards **1**–**8** (in this case, different cancer cell line could be used); and 2/ the complexes are unstable under the experimental condition used and thus unsuitable for further biological studies.

#### ^1^H NMR spectroscopy of complexes 2 and 8 in DMF-*d*_*7*_

The reason why a pure DMF-*d*_7_ was utilized for the stability study is that this solvent is typically used for the pre-dissolution (and consequently, the DMF-*d*_7_ solution is diluted by medium to a maximal DMF concentration of 0.1%) of the corresponding complex during the cytotoxicity testing. Logically, it was of a great interestto find out if the composition of the complexes can be affected by this solvent. The signals of C2–H hydrogen atom of the *n*aza ligands of **2** and **8** as well as aromatic hydrogens C13,15–H_2_ and C12,16–H_2_ of *p*-cymene were chosen as the representatives for such comparative study with the aim to show if the complexes decompose/solvolyse after dissolving in the solvent used.

**Fig 4:**
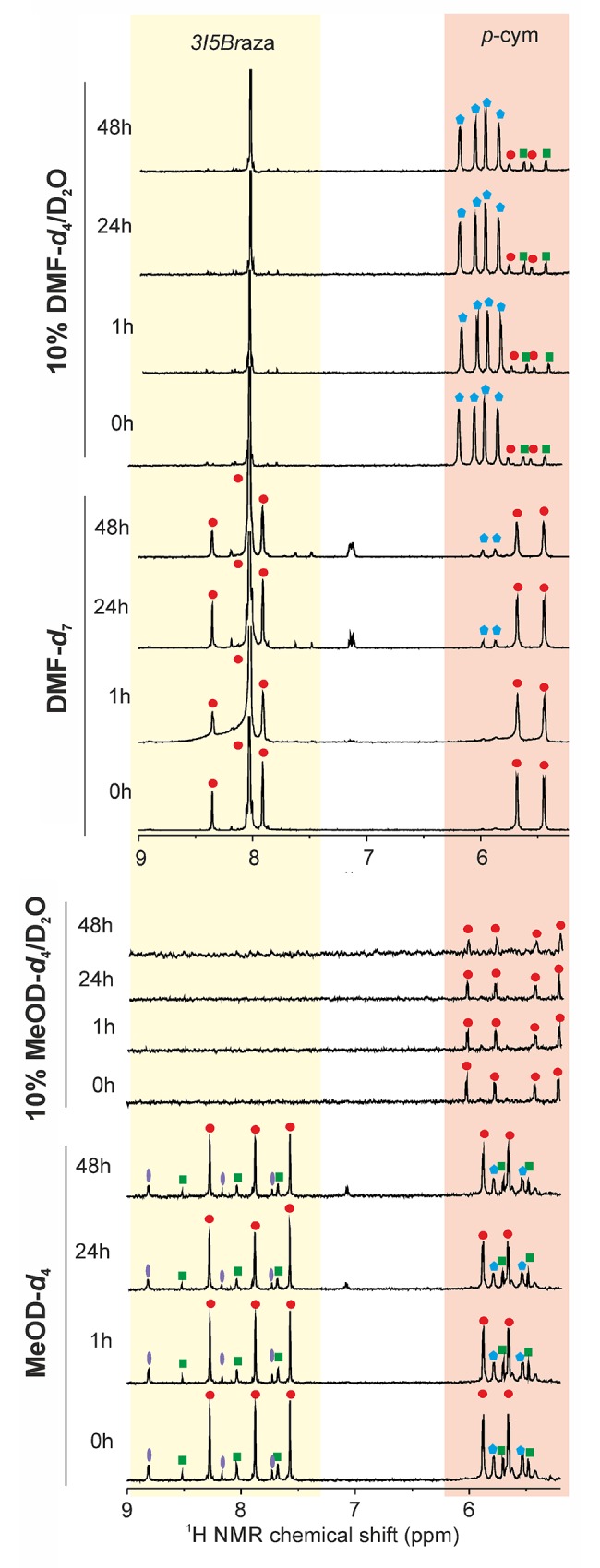
Selected ^1^H NMR spectra of complex 8 acquired on MeOD-*d*_*4*_, 10% MeOD-*d*_*4*_/D_2_O, DMF-*d*_*7*_ and 10% DMF-*d*_*7*_/D_2_O solutions at different time points (0 h, 1 h, 24 h or 48 h).

In the case of **2**, two sets of the C2–H/C13,15–H_2_/C12,16–H_2_ signals revealed in the ^1^H NMR spectrum of its fresh DMF-*d*_7_ solution at 7.80/5.88/5.66 and 7.75/5.78/5.49 ppm, and their integral intensity ratio of 1.0: 0.6 did not change in time up to 48 h (S4 Fig). Similarly, two sets of the mentioned signals were found in the fresh DMF-*d*_7_ NMR spectrum of **8** (Fig 4) at 7.92/5.68/5.65 and 7.48/5.99/5.87 ppm. The peaks revealed at 7.80 ppm (for **2**) and 7.92 ppm (for **8**) may be associated with the C2–H hydrogen atoms of the *n*aza ligands, while the peaks observed at 5.68/5.66 ppm (for **2**) and 5.68/5.65 ppm (for **8**) can be attributed to the aromatic hydrogens C13,15–H_2_, and C12,16–H_2_ of the coordinated *p*-cymene, both of them belonging to the original (i.e. unchanged) complexes **2** and **8**. The second sets of the signals attributable to the *n*aza and *p*-cymene ligands, which revealed at 7.75/5.78/5.49 ppm (for **2**) and 7.48/5.99/5.87 ppm (for **8**), may be associated with the formation of decomposition products, probably connected with the solvolysis (substitution of the chlorido ligand(s) with DMF). This finding is, in the case of **8**, supported by an increasing portion of this impurity from < 5% in the fresh DMF-*d*_7_ solution to ca 20% after 48 h (Fig 4).

#### ^1^H NMR spectroscopy and ESI mass spectrometry of complexes 2 and 8 in 10% DMF-*d*_*7*_/D_2_O

After the addition of D_2_O into the DMF-*d*_7_ solution of **2**, four pairs of signals belonging to C13,15–H_2_/C12,16–H_2_ were detected at 6.10/5.86, 6.23/5.97, 5.95/5.72 and 5.50/5.30 ppm, with the integral intensities being 1.00, 0.25, 0.36, and 0.30, respectively (S4 Fig, S5 Fig). A position of the doublets at 6.10/5.86 and 5.95/5.72 ppm correlated well with those detected in the spectrum of the starting ruthenium(II) dimer (S5 Fig), while the doublets at 6.23/5.97 and 5.50/5.30 ppm belong probably to species associated with the hydrolysis of **2** (see below). The release of *3Cl*aza from the structure of **2**, connected with the formation of the starting ruthenium(II) compound from **2** in 10% DMF-*d*_7_/D_2_O, was confirmed because the signal of C2–H belonging to free *3Cl*aza was detected at 7.63 ppm in the ^1^H NMR spectrum of **2** (a C2–H signal showed at the same position in the ^1^H NMR spectrum of free *3Cl*aza dissolved in 10% DMF-*d*_7_/D_2_O).

The mentioned C13,15–H_2_/C12,16–H_2_ doublets found at 6.23/5.97 and 5.50/5.30 ppm were consistent in the integral intensities with the C2–H signals at 7.70 and 7.74 ppm, indicating that both the mentioned set of signals belong to the species containing both *3Cl*aza and *p*-cymene ligands. The positions of these signals do not correlate with those detected in the DMF-*d*_7_ spectrum. Based on the discussed ^1^H NMR results it could be anticipated that the latter two set of signals most likely belong to the products of hydrolysis, such as probably {[Ru(*η* ^6^-*p*-cym)(*3Cl*aza)(H_2_O)Cl]}^+^ and/or {[Ru(*η* ^6^-*p*-cym)(*3Cl*aza)(H_2_O)_2_]}^2+^, and/or products of their protolysis involving the OH^−^ligands. To get an evidence for the formation of the mentioned species, ESI mass spectrometry experiments were performed on **2** dissolved in 10% DMF/H_2_O solution. The obtained spectrum of **2** recorded on the fresh solution in 10% DMF/H_2_O contained many peaks of ruthenium-containing species, including those of the species detected also in mass spectrum of **2** dissolved in MeOH, namely [Ru_2_(*η* ^6^-*p*-cym)_2_Cl_3_]^+^ (578.9 *m/*z), [Ru(*η* ^6^-*p*-cym)(*3Cl*aza)Cl]^+^ (422.9 *m/z*), {[Ru(*η* ^6^-*p*-cym)(*3Cl*aza)]–H}^+^ (387.0 *m/z*), [Ru(*η* ^6^-*p*-cym)Cl]^+^ (271.0 *m/z*) and {(*3Cl*aza)+H}^+^ (153.1 *m/z*). Apart from those, several new peaks showed in the spectrum of **2** in 10% DMF/H_2_O as compared with the spectrum of **2** in MeOH. In particular, overlapped peaks of [Ru_2_(*η* ^6^-*p*-cym)_2_(OH)Cl_2_]^+^ and [Ru_2_(*η* ^6^-*p*-cym)_2_(OH)_3_Cl]^+^ (10:1 ratio; 558.0 *m/*z), and the peaks of the {[Ru_2_(*η* ^6^-*p*-cym)_2_Cl]+O}^+^ (522.1 *m/*z), [Ru(*η* ^6^-*p*-cym)(DMF)Cl]^+^ (343.9 *m/z*) and [Ru(*η* ^6^-*p*-cym)(H_2_O)Cl]^+^ (288.9 *m/z*) species were observed in the spectra. To sum up, although the above mentioned hydrolytic products, e.g. {[Ru(*η* ^6^-*p*-cym)(*3Cl*aza)(H_2_O)Cl]}^+^ and/or {[Ru(*η* ^6^-*p*-cym)(*3Cl*aza)(H_2_O)_2_]}^2+^, were not detected, it is evident from the ESI+ mass spectrometry results that the complex **2** is decomposed to various species.

Addition of D_2_O into the DMF-*d*_7_ solution of **8** led to the formation of white precipitate which was centrifuged, and the isolated solid was dissolved in DMF-*d*_7_ and proved to be free *3I5Br*aza by ^1^H NMR experiment. After removing of the precipitate, the ^1^H NMR spectrum of **8** showed, in total, four pairs of doublets belonging to C13,15–H_2_/C12,16–H_2_ detected at 5.51/5.31, 5.64/5.44, 5.95/5.75 and 6.10/5.87 ppm, with the integral intensities being 0.18, 0.15, 1.00, and 1.00, respectively (Fig 4). No *3I5Br*aza signals were found in the appropriate proton spectra. In other words, addition of D_2_O into the DMF-*d*_7_ solution of **8** led to its complete decomposition connected with a release of *3I5Br*aza (Fig 4). Similarly to **2**, the mass spectrum of **8** dissolved in 10% DMF/H_2_O revealed [Ru_2_(*η* ^6^-*p*-cym)_2_Cl_3_]^+^, [Ru(*η* ^6^-*p*-cym)Cl]^+^, the overlapped peaks of [Ru_2_(*η* ^6^-*p*-cym)_2_(OH)Cl_2_]^+^ and [Ru_2_(*η* ^6^-*p*-cym)_2_(OH)_3_Cl]^+^, {[Ru_2_(*η* ^6^-*p*-cym)_2_Cl]+O}^+^, [Ru(*η* ^6^-*p*-cym)(DMF)Cl]^+^ and [Ru(*η* ^6^-*p*-cym)(H_2_O)Cl]^+^ (288.9 *m/z*), indicating analogical behaviour of both the studied complexes **2** and **8** in the used mixture of solvents.

Generally said, dissolving of the studied complexes in 10% DMF-*d*_7_/D_2_O (or 10% DMF/H_2_O) led to the release of *n*aza and the formation of low active or most probably non-potent ruthenium-containing species. Since the used mixture of solvents was similar to that one used for *in vitro* cytotoxicity testing (0.1% DMF in RPMI-1640 medium), it can be anticipated that similar processes proceeded within the performed biological testing, altogether resulting in inactivity of the studied complexes against the used human cancer cell line.

#### ^1^H NMR spectroscopy of complexes 2 and 8 in MeOD-*d*_*4*_

Since it was observed that the studied complexes are stable in CDCl_3_ (see section Chemistry, Fig 2, with a coordination ability index of –2.2) and unstable in DMF-*d*_7_ (see above, with a coordination ability index of –0.2), as judged by ^1^H NMR spectra, we strived to investigate their solution behaviour also in another relevant solvent (i.e. methanol), with a coordination ability of –0.4 [34].

The ^1^H NMR spectra of **2** and **8** recorded on MeOD-*d*_*4*_ solutions contained two sets of signals (according to their integral intensities), represented by C2–H/C13,15–H_2_/C12,16–H_2_ signals detected at 7.53/5.78/5.50 and 7.58/5.88/5.66 ppm for **2** (S4 Fig) and at 7.55/5.87/5.66 and 7.79/5.62/5.49 ppm for **8** (Fig 4). These signals pointed out the fact that both the *p*-cymene and *n*aza ligands are present in both the complex species. Moreover, another set of the *n*aza signals was also found in the spectra of both **2** and **8**. The position of the C2–H signal is consistent with the free *3Cl*aza, as proved for **2** (7.42 ppm). However, the same could not be proved in the case of complex **8** because it contains *3I5Br*aza which is insoluble in methanol. This indicated that the mentioned set of the *3I5Br*aza signals detected in the MeOD-*d*_*4*_ solution of **8** most probably did not belong to a released *3I5Br*aza but to a complex containing the mentioned ligand. Moreover, in the case of **8**, another pair of *p*-cymene aromatic hydrogen atom signals (i.e. C13,15–H_2_/C12,16–H_2_) was observed at 5.79/5.54 ppm, but it was not accompanied by the appropriate signals (C2–H) at the *3I5Br*aza region (Fig 4).

Overall, the individual signals of the spectra as well as their integral intensities did not change in time up to 48 h of standing at ambient temperature for both **2** and **8** (Fig 4, S4 Fig). Thus, it could be concluded that at least a part of the complexes **2** and **8** decomposed in MeOD-*d*_*4*_ together with the release of *n*aza, which correlates well with the mass spectrometry results obtained for MeOH solutions of the studied complexes, as discussed above.

#### ^1^H NMR spectroscopy and ESI mass spectrometry of complexes 2 and 8 in 10% MeOD-*d*_*4*_/D_2_O

The studied complex **2** showed six pairs of C13,15–H_2_/C12,16–H_2_ signals at 6.16/5.87, 6.04/5.79, 5.97/5.83, 5.91/5.68, 5.65/5.23 and 5.58/5.44 ppm, and three sets of signals in the region of 7-azaindole aromatic hydrogens of *3Cl*aza represented by C2–H singlets at 7.67, 7.62 and 7.60 ppm (S4 Fig), but the position of none of these C2–H signals did not correlate with the signals of free (i.e. released) *3Cl*aza in 10% MeOD-*d*_*4*_/D_2_O.

As in the case of DMF-*d*_7_/D_2_O solution, the addition of D_2_O into the MeOD-*d*_*4*_ solution of **8** resulted to the formation of white precipitate, which was centrifuged before the ^1^H NMR acquisition. The spectra of **8** recorded on 10% MeOD-*d*_*4*_/D_2_O solutions contained only the signals of the *p*-cymene hydrogens, represented by two pairs of C13,15–H_2_/C12,16–H_2_ signals at 5.98/5.74 and 5.38/5.17 ppm (an integral intensity ratio of 1: 1) (Fig 4). Both these pairs of doublets correlated with those of the starting ruthenium(II) dimer in 10% MeOD-*d*_*4*_/D_2_O (an integral intensity ratio of 1.0: 0.2). On the other hand, any C2–H signals were not detected at the *3I5Br*aza region for **8**.

It can be concluded that, as in the case of 10% DMF-*d*_7_/D_2_O, **2** and **8** rapidly decompose/solvolyse in the used 10% MeOD-*d*_*4*_/D_2_O solution, which is in the case of **8** provably connected with a release of *3I5Br*aza. The solvolysis (most probably hydrolysis) of **2** and **8** was indicated also by ESI+ mass spectrometry utilizing the samples dissolved in 10% MeOH/H_2_O solution. As can be seen from S2 Fig and S6 Fig, the mass spectra of **2** dissolved both in MeOH and 10% MeOH/H_2_O contained the overlapped peaks of {[Ru_2_(*η* ^6^-*p*-cym)_2_(OCH_3_)]+2H}^+^ and {[Ru_2_(*η* ^6^-*p*-cym)_2_Cl]+2H}^+^ (1:1 ratio; 507.0 *m/*z), [Ru_2_(*η* ^6^-*p*-cym)_2_Cl_2_]^+^ and [Ru_2_(*η* ^6^-*p*-cym)_2_(OCH_3_)Cl]^+^ (5:1 ratio; 542.0 *m/*z), and [Ru_2_(*η* ^6^-*p*-cym)_2_Cl_3_]^+^, [Ru_2_(*η* ^6^-*p*-cym)_2_(OCH_3_)Cl_2_]^+^ and [Ru_2_(*η* ^6^-*p*-cym)_2_(OCH_3_)_2_Cl]^+^ (2:1:2 ratio; 570.9 *m/*z), as well as the peaks of [Ru(*η* ^6^-*p*-cym)(*3Cl*aza)Cl]^+^ (422. *η* ^6^-*p*-cym)(*3Cl*aza)]–H}^+^ (387.0 *m/z*), [Ru(*η* ^6^-*p*-cym)Cl]^+^ (271.0 *m/z*) and {(*3Cl*aza)+H}^+^ (153.1 *m/z*). However, some additional peaks were also detected in the spectra of **2** in 10% MeOH/H_2_O solution as compared with the spectra detected in the MeOH solution. The additional peaks most probably belong, according to their mass and isotopic patterns, to [Ru_2_(*η* ^6^-*p*-cym)_2_(OH)]^+^ (488.1 *m/*z), overlapped [Ru_2_(*η* ^6^-*p*-cym)_2_(OCH_3_)(O)]^+^ and [Ru_2_(*η* ^6^-*p*-cym)_2_Cl(O)]^+^ (1:1 ratio; 519.0 *m/*z), and {[Ru_2_(*η* ^6^-*p*-cym)_2_Cl_2_(O_2_)]+2H}^+^ (577.0 *m/*z) (S6 Fig). No changes were found in the spectrum recorded after 24 and 48 h of standing at ambient temperature. Similarly, the additional peaks of the [Ru_2_(*η* ^6^-*p*-cym)_2_(OH)]^+^ (488.1 *m/*z), [Ru_2_(*η* ^6^-*p*-cym)_2_(OCH_3_)]^+^ (503.0 *m/*z), [Ru_2_(*η* ^6^-*p*-cym)_2_(OCH_3_)(O)]^+^ (518.9 *m/*z), [Ru_2_(*η* ^6^-*p*-cym)_2_(OCH_3_)_3_]^+^ (565.0 *m/*z) and {(*3I5Br*aza)+H}^+^ (322.8 *m/z*) species were detected in the fresh 10% MeOH/H_2_O solution of **8** with no change after 24 and 48 h.

The obtained results showed that additionally to the peaks detected in the mass spectra of MeOH solution of **2** or **8**, several new peaks were found after the addition of water (S6 Fig). As it is clearly seen from the anticipated composition of the new species detected on the water-containing solution of the representative complexes, their formation is closely related to the interaction with water (hydrolysis) and release of *N*-donor *n*aza ligands.

#### ^1^H NMR studies of interactions of 2 and 8 with reduced glutathione (GSH)

^1^H NMR spectra recorded on 10% MeOD-*d*_*4*_/D_2_O solutions of the mixture of **2** and GSH contain three pairs of C13,15–H_2_/C12,16–H_2_ signals of *p*-cymene and one set of the C2–H signals of *3Cl*aza with no correlation in integral intensity. The position of the representative C2–H signal of *3Cl*aza observed in the 10% MeOD-*d*_*4*_/D_2_O solution correlated with that of free *3Cl*aza dissolved in the same mixture of solvents, indicating that *3Cl*aza released from the structure of **2**. As for the GSH region, two triplets of Cys-*α* CH were detected at 4.49 and 4.55 ppm and two Cys-*β* CH_2_ signals were found at 2.77 and 2.86 ppm, with an integral intensity of signals at 4.49/2.86 ppm versus those at 4.55/2.77 ppm being ca 1: 9. A set of signals represented by Cys-*α*/Cys-*β* pair of signals at 4.49/2.86 ppm belongs to free GSH, as proved by ^1^H NMR experiment carried out for GSH dissolved in the used 10% MeOD-*d*_*4*_/D_2_O solutions, correlating with the literature data [35]. With respect to the above mentioned findings, it is evident that most of the studied complex **2** decomposed leading to the formation of an Ru–containing adduct with the deprotonated glutathione coordinated through the S-atom (Ru–SG). An integral intensity of the signals of the Ru–SG adduct was not consistent with the above mentioned *p*-cymene signals, proving that the Ru–SG adduct does not contain neither *p*-cymene nor *3Cl*aza ligand. Interestingly, a ratio of Cys-*α* and Cys-*β* signals of free and coordinated glutathione (1: 9 for the fresh solution, as mentioned above) changed to ca 1: 1 after 48 h of standing at the ambient temperature, meaning that the formed Ru–SG adduct is unstable in the used mixture of solvents, which is connected with a release of GSH.

^1^H NMR spectra recorded on 10% MeOD-*d*_*4*_/D_2_O solutions of the mixture of **8** with GSH did not contain any signals in the *n*aza and *p*-cymene region and only the signals of the used biomolecules were detected. The set of signals, detected at 4.49/2.86 ppm and 4.55/2.77 ppm ppm for **2**, were also detected in the spectra of the fresh solution of **8** in 10% MeOD-*d*_*4*_/D_2_O. However, an integral intensity ratio (9: 1) was found to be inverse as compared with **2**, and did not change in time, which showed that equilibrium between free GSH and Ru–SG adduct is reached rapidly. Again, the Ru–SG adduct does not contain neither *3Cl*aza nor *p*-cymene ligands.

The results of ^1^H NMR studies proved that interactions of **2** and **8** with GSH led to the formation of Ru–SG adduct and to a complete release of *n*aza from the structures of **2** and **8**. These results are consistent with a detection of various adducts with GSH in the ESI+ mass spectra of the studied complexes dissolved in 10% MeOH/H_2_O. Concretely, the peaks, whose mass and isotopic pattern correspond to [Ru(*η* ^6^-*p*-cym)(GS)]^+^ (542.1 *m/z*), {[Ru_2_(*η* ^6^-*p*-cym)_2_(GS)_2_]–H}^+^ (1082.7 *m/z*) and [Ru_2_(*η* ^6^-*p*-cym)_2_(GS)_3_]^+^ (1390.2 *m/z*) formed by an interaction of GSH with **2** (S7 Fig), were detected. Surprisingly, the analogical ESI+ mass spectrometry experiments performed for **8** led to the finding that this technique is not suitable for the solution of this issue because the spectra revealed no peaks associated that the adduct containing Ru–SG species.

#### Fluorescence quenching experiments

It is well-known that intercalative binding of EtBr to DNA leads to fluorescence emission, which could be quenched by replacement of EtBr from the mentioned EtBr/DNA adducts by various agents (e.g. transition metal complexes) [36,37]. On the other hand, 7-azaindole and its derivatives are known fluorescence emitting agents themselves [38–40]. Being aware of these facts and the above discussed results of ^1^H NMR spectroscopy and ESI+ mass spectrometry indicating a release of the *n*aza ligands from the structures of the studied complexes, it was of great interest to perform the fluorescence quenching experiments on EtBr/ctDNA in the presence of the selected representatives of **1**–**8**.

**Fig 5:**
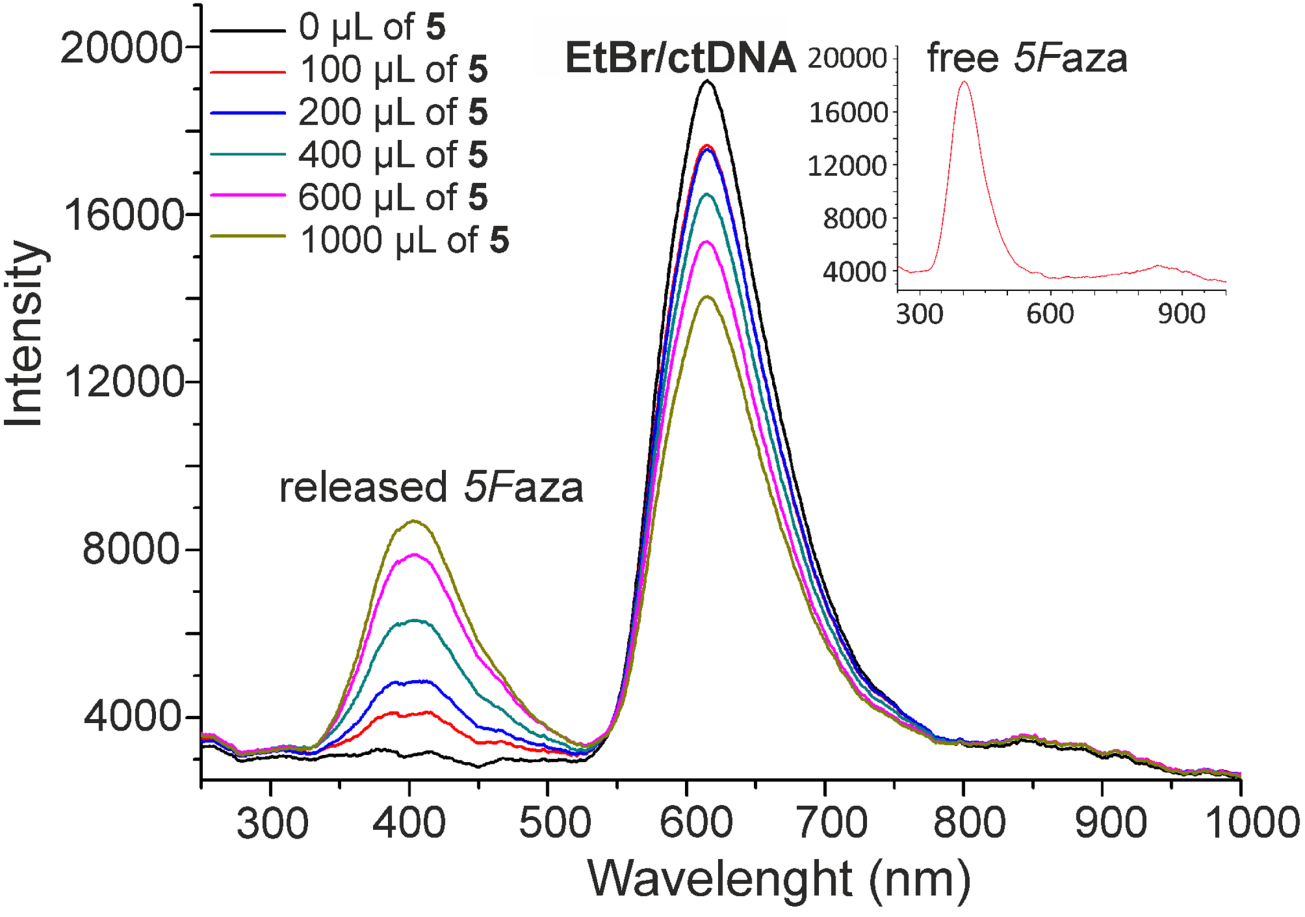
Fluorescence quenching curves of EtBr/ctDNA adduct with the complex [Ru(*η* ^6^-*p*-cym)(*5F*aza)Cl_2_] (5). The depicted curves were recorded after addition of various volumes of 150 μM stock solution of **5**. A fluorescence spectrum of free *5F*aza is inserted.

After the addition of the representative Ru(II) complexes **2**, **5** or **8**, the concentration-dependent fluorescence quenching of EtBr was observed, showing on the ability of the studied complexes or their decomposition/solvolysis product/s to interact with the used ctDNA (Fig 5 for **5**); *note*: **5** was used together with the representative complexes **2** and **8**, because *5F*aza contained in its structure showed markedly higher fluorescence as compared with *3Cl*aza (contained in **2**; lower fluorescence) and *3I5Br*aza (contained in **8**; no fluorescence because of limited solubility in the medium used). Except the fluorescence maximum of EtBr/ctDNA adduct detected at 615 nm, another peak showed in the obtained fluorescence spectra of **2** (at 415 nm) and **5** (at 405 nm; Fig 5). The position of this peak corresponds to that of free *n*aza (inset in Fig 5), which proved, as anticipated, a release of the *n*aza ligands.

## Conclusions

The organometallic [Ru(*η* ^6^-*p*-cym)(*n*aza)Cl_2_] complexes (**1**–**8**; *p*-cym = *p*-cymene; *n*aza = 7-azaindole or its derivatives) were prepared and thoroughly characterized by relevant techniques including a crystallographic study of [Ru(*η* ^6^-*p*-cym)(*2Me4Cl*aza)Cl_2_] (**6**) showing a piano-stool arrangement with *N*-donor ligand coordination through its *N*7 atom. The complexes were studied for their anticancer activity against the A2780 human cancer cell line, however, no cytotoxicity was found up to the tested concentration of 50 μM. That is why we were looking for the reason of the inactivity. Thus, the complexes were studied for their stability in various solvents (MeOH, DMF, and their mixtures with water). These studies revealed the complexes to be highly unstable, because besides a rapid solvolysis they decompose to the starting ruthenium(II) compound, [Ru(μ-Cl)(*η* ^6^-*p*-cym)Cl]_2_ and/or its solvolysis products, which is connected with a release of *n*aza. With respect to instability of the studied complexes in the solvents used, it may be concluded that the anticancer inactivity of the compounds is associated with this property and formation of inactive species (starting ruthenium(II) dimer, released *n*aza, solvolysis products). It has to be noted that such findings are rather unexpected because the literature data clearly reveal that complexes of a general formula [Ru(*η* ^6^-*p*-cym)(L)Cl_2_] should show anticancer activity, as can be seen from references [14–17] reporting the complexes of the mentioned general formula potent against A2780 ovarian carcinoma cells used also in this work.

## Supporting Information

S1 Fig Experimental (up) and simulated (down) ESI+ mass spectrum of the [Ru(*η* ^6^-*p*-cym)(*3I5Br*aza)Cl]^+^ species. ESI+ mass spectrum was recorded at 100–800 *m/z* on the MeOH solution of **8.**
(TIF)

S2 Fig ESI+ mass spectra (150–700 *m/z* range) of the methanolic solutions of the starting ruthenium(II) dimer [Ru(μ-Cl)(*η* ^6^-*p*-cym)Cl]_2_ (down) and the studied complex 8 (up). (TIF)

S3 Fig Part of the crystal structure of [Ru(*η* ^6^-*p*-cym)(*2Me4Cl*aza)Cl_2_] (6). The drawing shows the formation of supramolecular 3D structure through the selected C12–H12···C6, C13–H13···C19, C13···Cl1, C16–H16···Cl2, C18–H18A···C5 and C20–H20A···C16 non-covalent contacts (red dashed lines); symmetry codes: ii) 1.5-x, y-0.5, 0.5-z; iii) x, y-1, z; iv) 1-x, 1-y, -z; v) 1-x, -y, -z; vi) 1.5-x, y+0.5, 0.5-z; vii) x, y+1, z. The hydrogen atoms not involved in the depicted non-covalent contacts were omitted for clarity.
(TIF)

S4 Fig Selected ^1^H NMR spectra of 2. The spectra were acquired on MeOD-*d*_*4*_, 10% MeOD-*d*_*4*_/D_2_O, DMF-*d*_*7*_ or 10% DMF-*d*_*7*_/D_2_O solutions of **2** at different time points (0 or 48 h). The peaks assigned with the same symbols have the same integral intensity.
(TIF)

S5 Fig ^1^H NMR spectra acquired on 10% DMF-*d*_*7*_/D_2_O solutions of the starting ruthenium(II) dimer [Ru(μ-Cl)(*η* ^6^-*p*-cym)Cl]_2_ (down), the studied complex [Ru(*η* ^6^-*p*-cym)(*3Cl*aza)Cl_2_] (2; middle) and free 3-chloro-7-azaindole (*3Cl*aza; up). (TIF)

S6 Fig ESI+ mass spectra of [Ru(*η* ^6^-*p*-cym)(*3Cl*aza)Cl_2_] (2). The spectrum (depicted at 400–600 *m/z* range) was acquired on the 10% MeOH/H_2_O solutions of **2** (up) and is given together with the details of the [Ru_2_(*η* ^6^-*p*-cym)_2_(OH)]^+^ and overlapped [Ru_2_(*η* ^6^-*p*-cym)_2_(OCH_3_)(O)]^+^ and [Ru_2_(*η* ^6^-*p*-cym)_2_Cl(O)]^+^ (1:1 ratio) species (middle) and their calculated simulations (down).
(TIF)

S7 Fig Experimental (up) and calculated (down) ESI+ mass spectra of the {[Ru(*η* ^6^-*p*-cym)(GSH)]–H}^+^ species. The spectrum was acquired on the 10% MeOH/H_2_O solution of **2** mixed with one molar equivalent of GSH.
(TIF)

S8 Fig Schematic representation of the *n*aza release from the structure of the studied complexes 1–8 as proved by the herein reported ^1^H NMR spectroscopy, ESI+ mass spectrometry and fluorescence quenching experiments. (TIF)

S1 Table Crystal data and structure refinement for [Ru(*η* ^6^-*p*-cym)(*2Me4Cl*aza)Cl_2_] (6) (PDF)

S2 Table Selected bond lengths (Å) and angles (°) of non-covalent contacts detected in the crystal structure of [Ru(*η* ^6^-*p*-cym)(*2Me4Cl*aza)Cl_2_] (6). (PDF)

S1 Text The results of FTIR spectroscopy and ESI+ mass spectrometry of 1–8. (PDF)
