# Supplementary figures and images for: Organometallic Half-Sandwich Dichloridoruthenium(II) Complexes with 7-Azaindoles: Synthesis, Characterization and Elucidation of Their Anticancer Inactivity against A2780 Cell Line
Source: PLoS One. 2015 Nov 25;10(11):e0143871. doi: 10.1371/journal.pone.0143871 (PMC4659567; doi:10.1371/journal.pone.0143871)

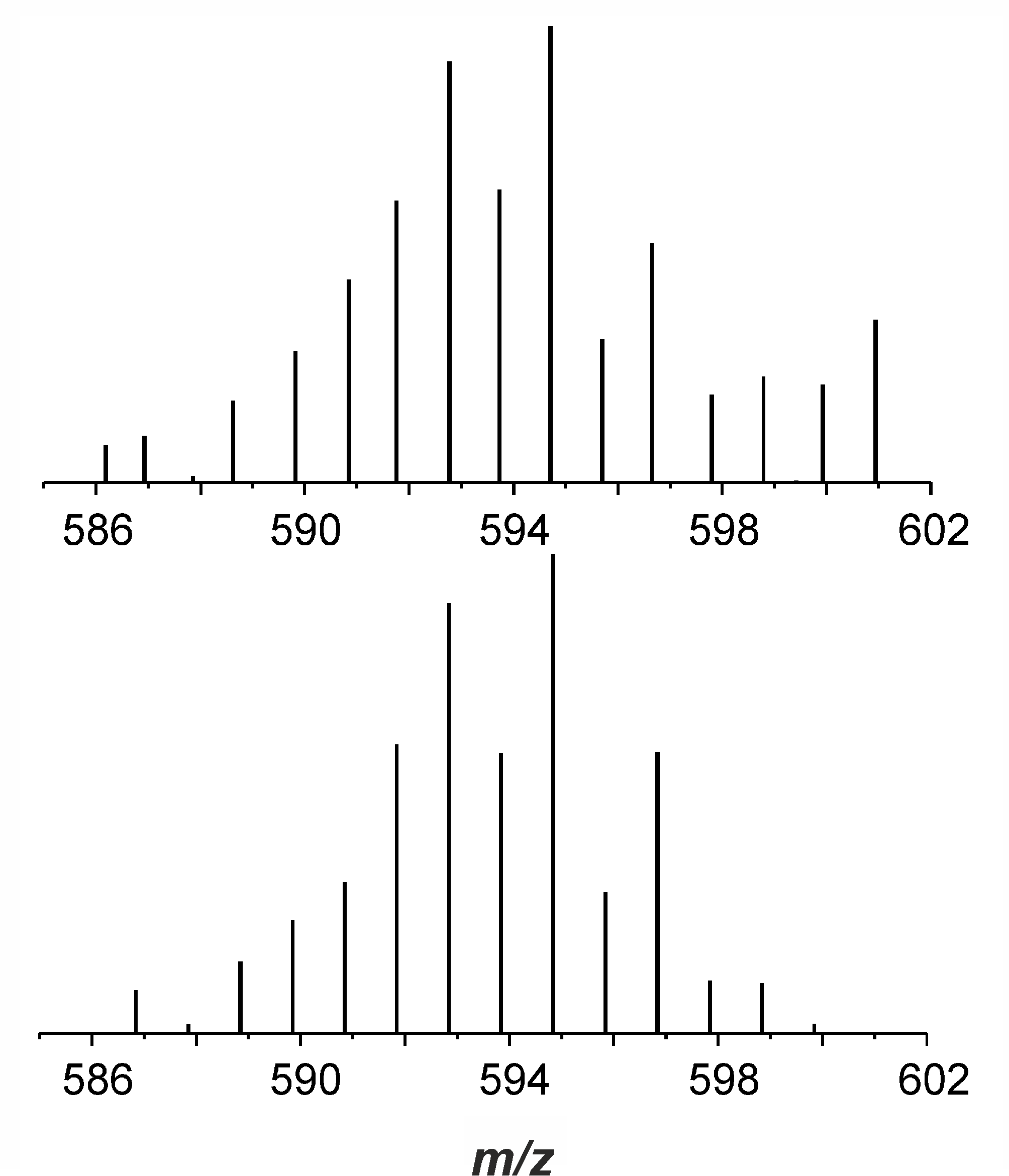

Supplement: S1 Fig — ESI+ mass spectrum was recorded at 100–800 m/z on the MeOH solution of 8. (TIF) [file pone.0143871.s001.tif]

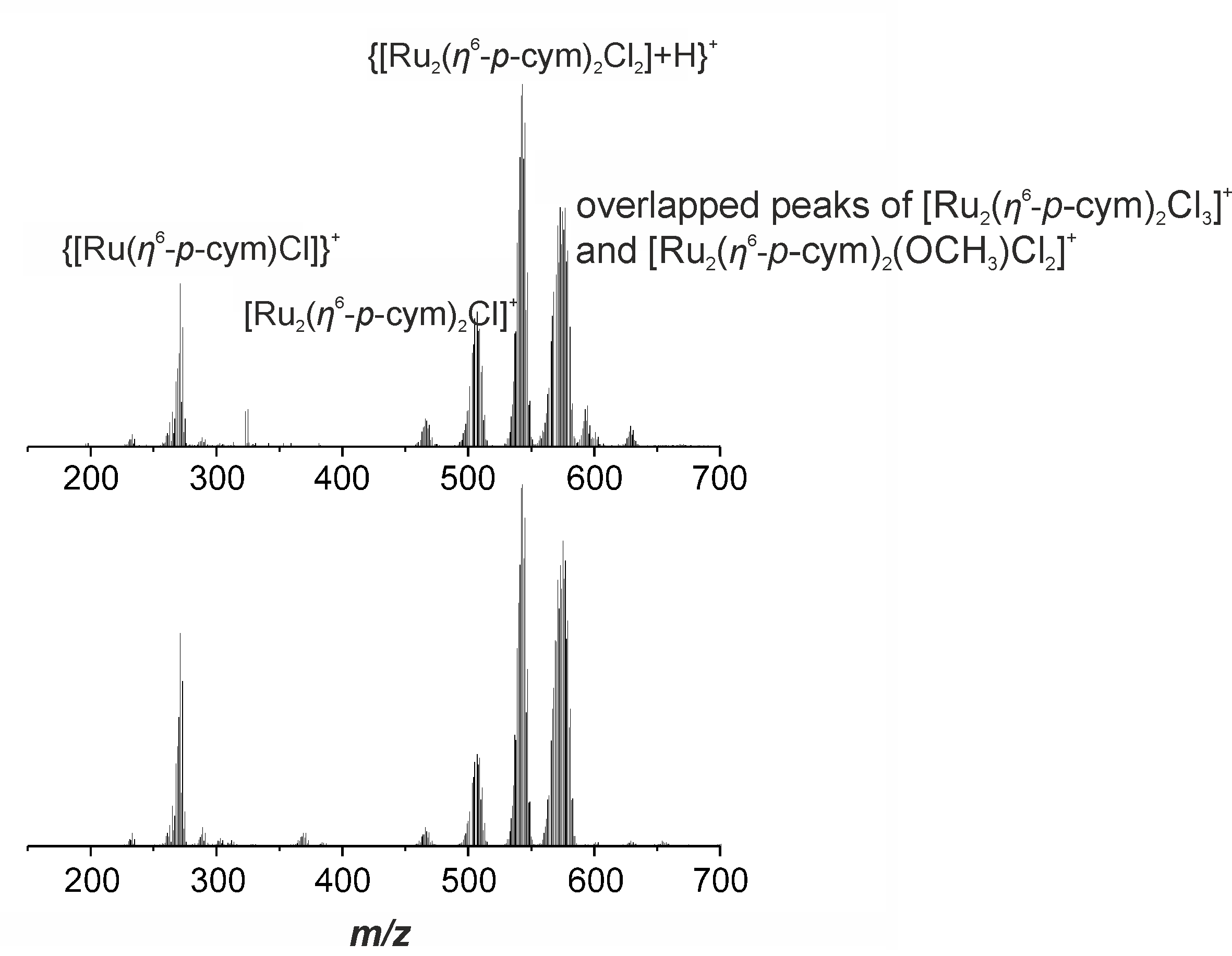

Supplement: S2 Fig — (TIF) [file pone.0143871.s002.tif]

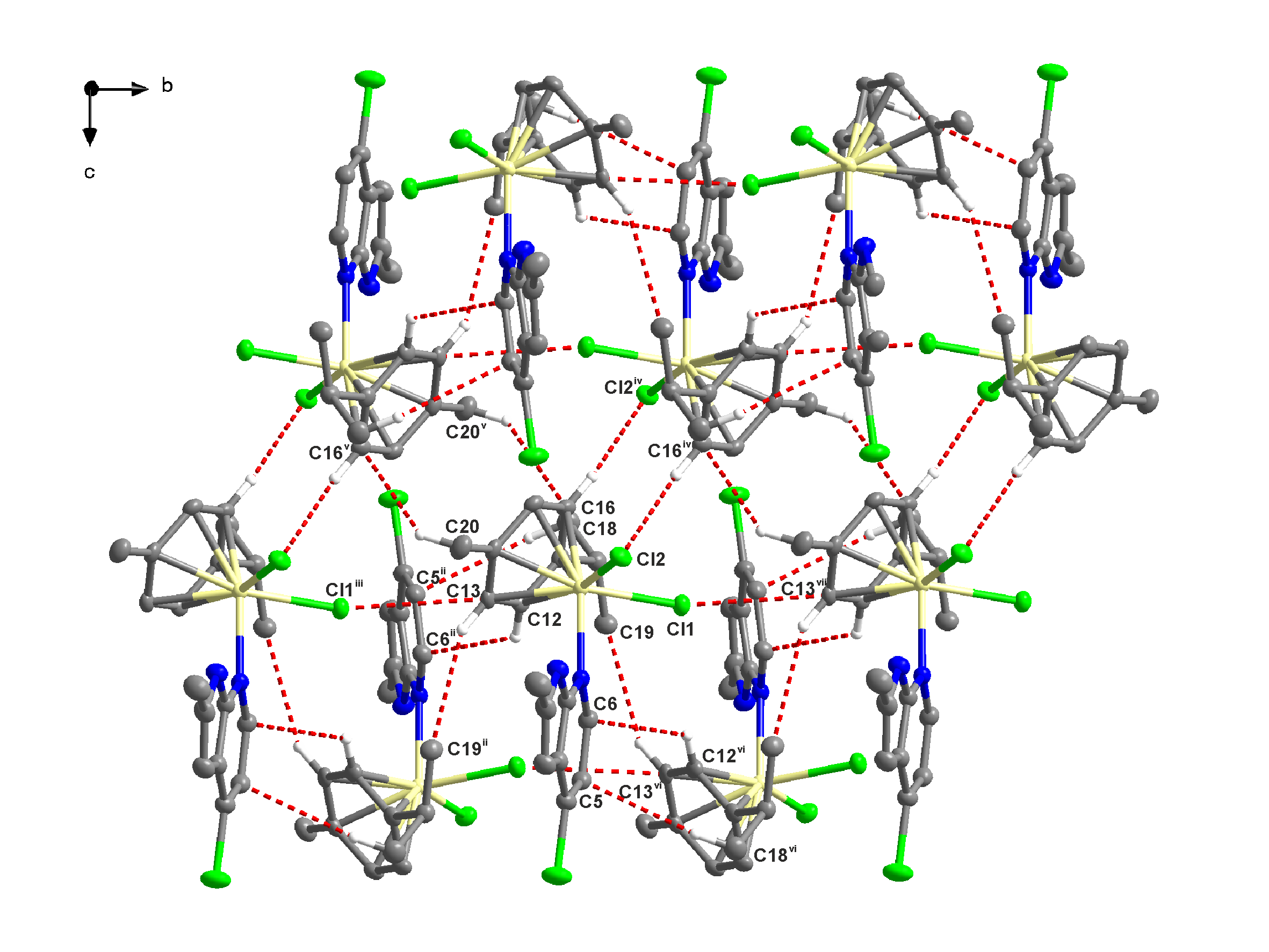

Supplement: S3 Fig — The drawing shows the formation of supramolecular 3D structure through the selected C12–H12···C6, C13–H13···C19, C13···Cl1, C16–H16···Cl2, C18–H18A···C5 and C20–H20A···C16 non-covalent contacts (red dashed lines); symmetry codes: ii) 1.5-x, y-0.5, 0.5-z; iii) x, y-1, z; iv) 1-x, 1-y, -z; v) 1-x, -y, -z; vi) 1.5-x, y+0.5, 0.5-z; vii) x, y+1, z. The hydrogen atoms not involved in the depicted non-covalent contacts were omitted for clarity. (TIF) [file pone.0143871.s003.tif]

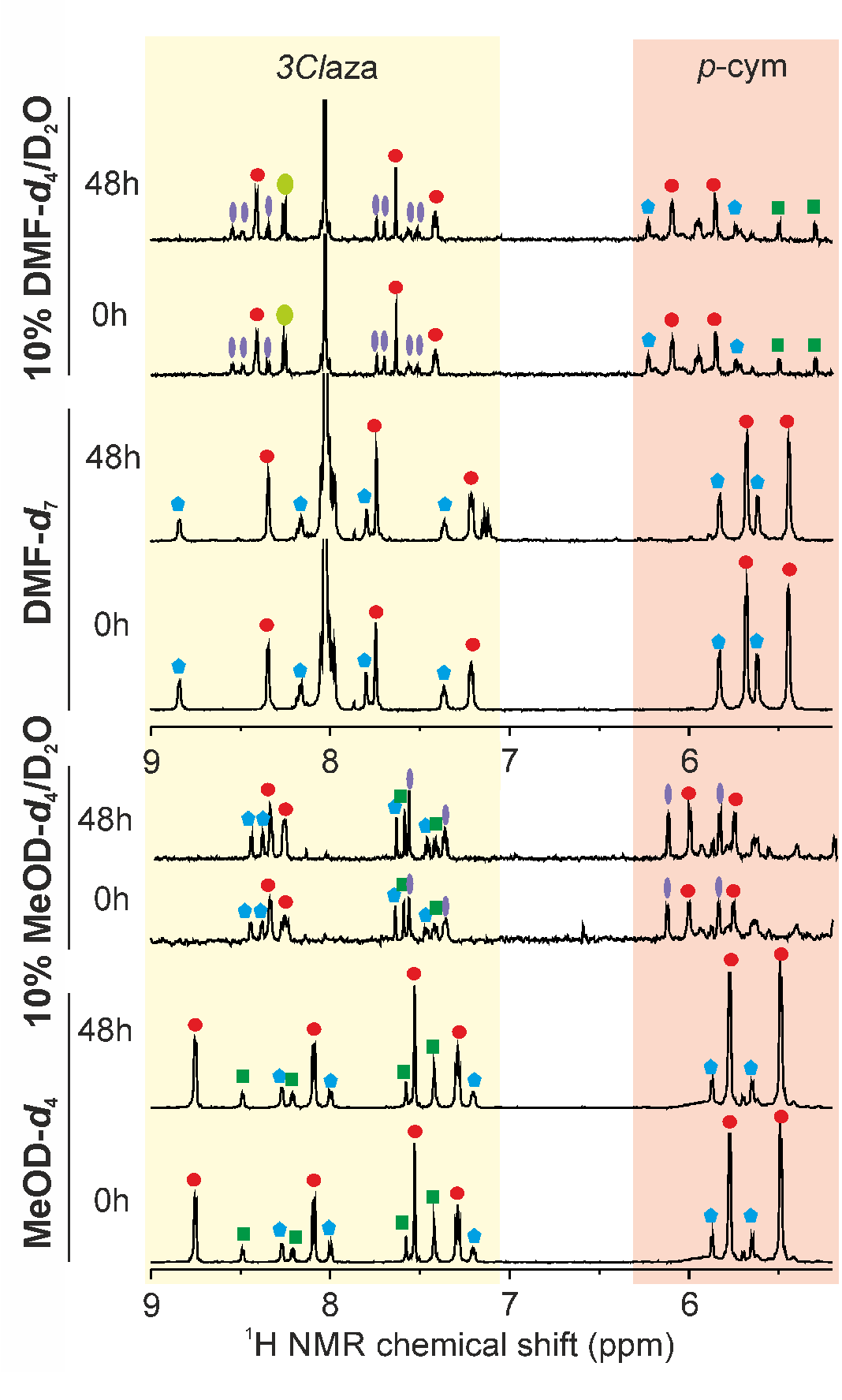

Supplement: S4 Fig — The spectra were acquired on MeOD-d 4, 10% MeOD-d 4/D2O, DMF-d 7 or 10% DMF-d 7/D2O solutions of 2 at different time points (0 or 48 h). The peaks assigned with the same symbols have the same integral intensity. (TIF) [file pone.0143871.s004.tif]

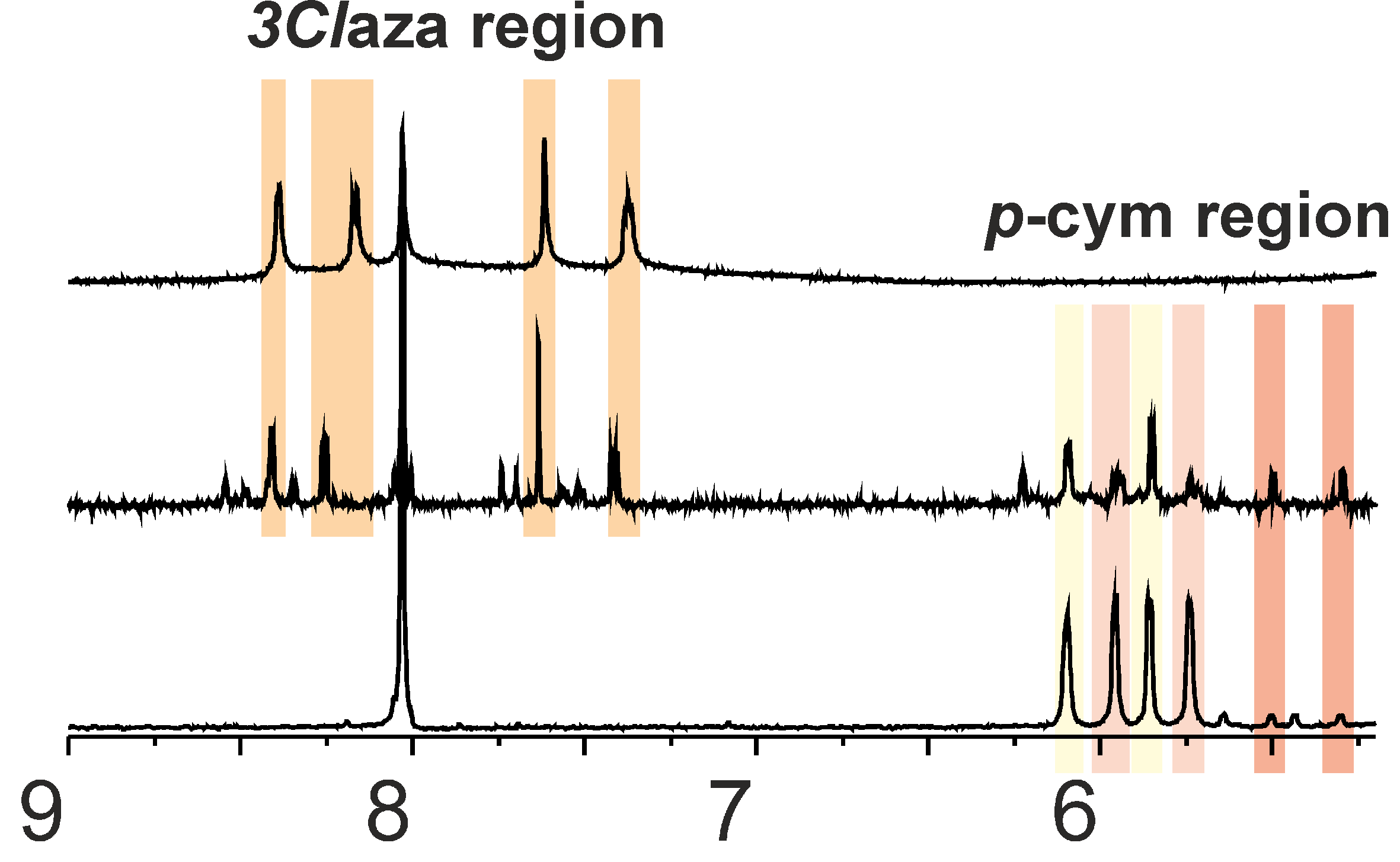

Supplement: S5 Fig — (TIF) [file pone.0143871.s005.tif]

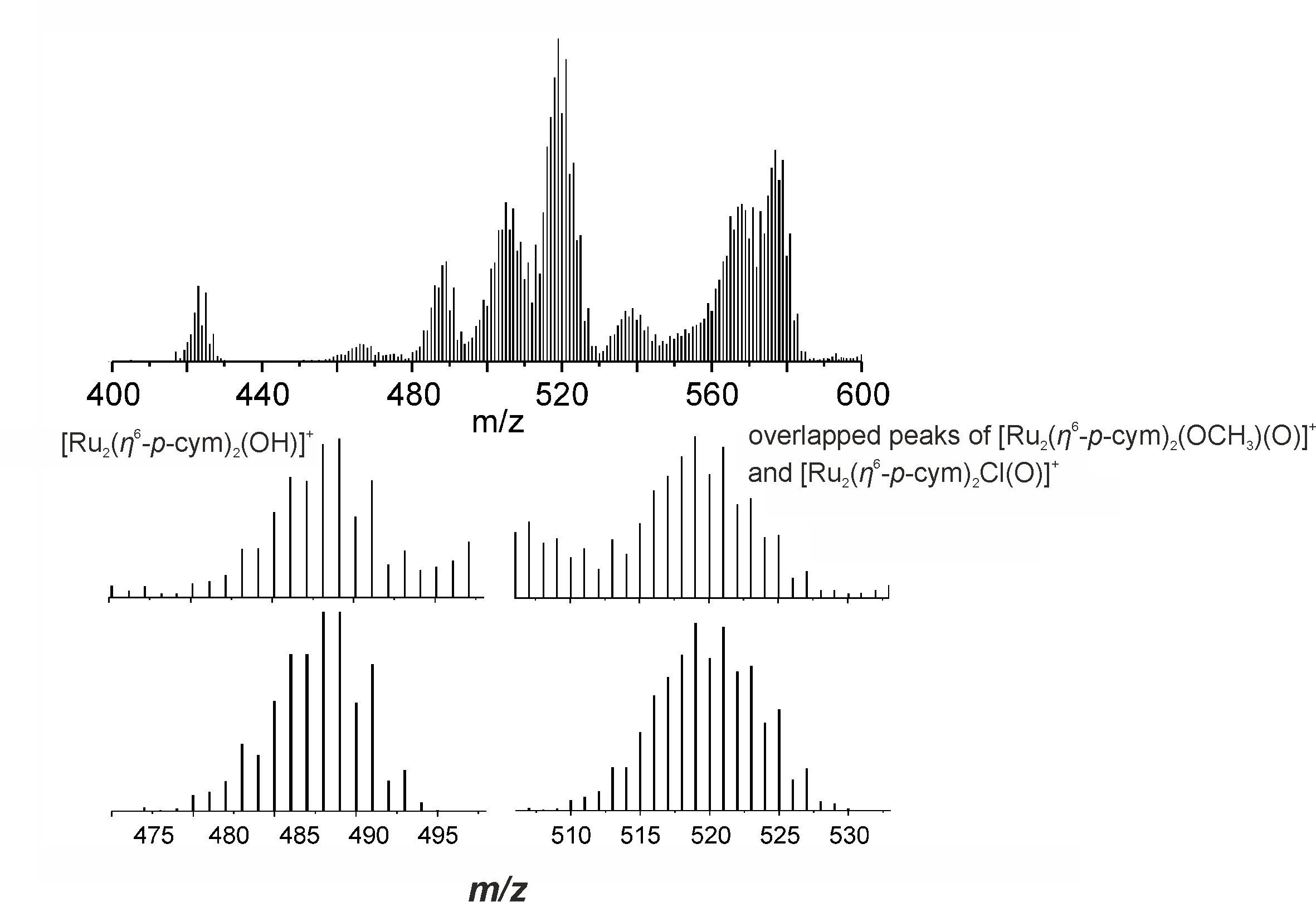

Supplement: S6 Fig — The spectrum (depicted at 400–600 m/z range) was acquired on the 10% MeOH/H2O solutions of 2 (up) and is given together with the details of the [Ru2(η 6-p-cym)2(OH)]+ and overlapped [Ru2(η 6-p-cym)2(OCH3)(O)]+ and [Ru2(η 6-p-cym)2Cl(O)]+ (1:1 ratio) species (middle) and their calculated simulations (down). (TIF) [file pone.0143871.s006.tif]

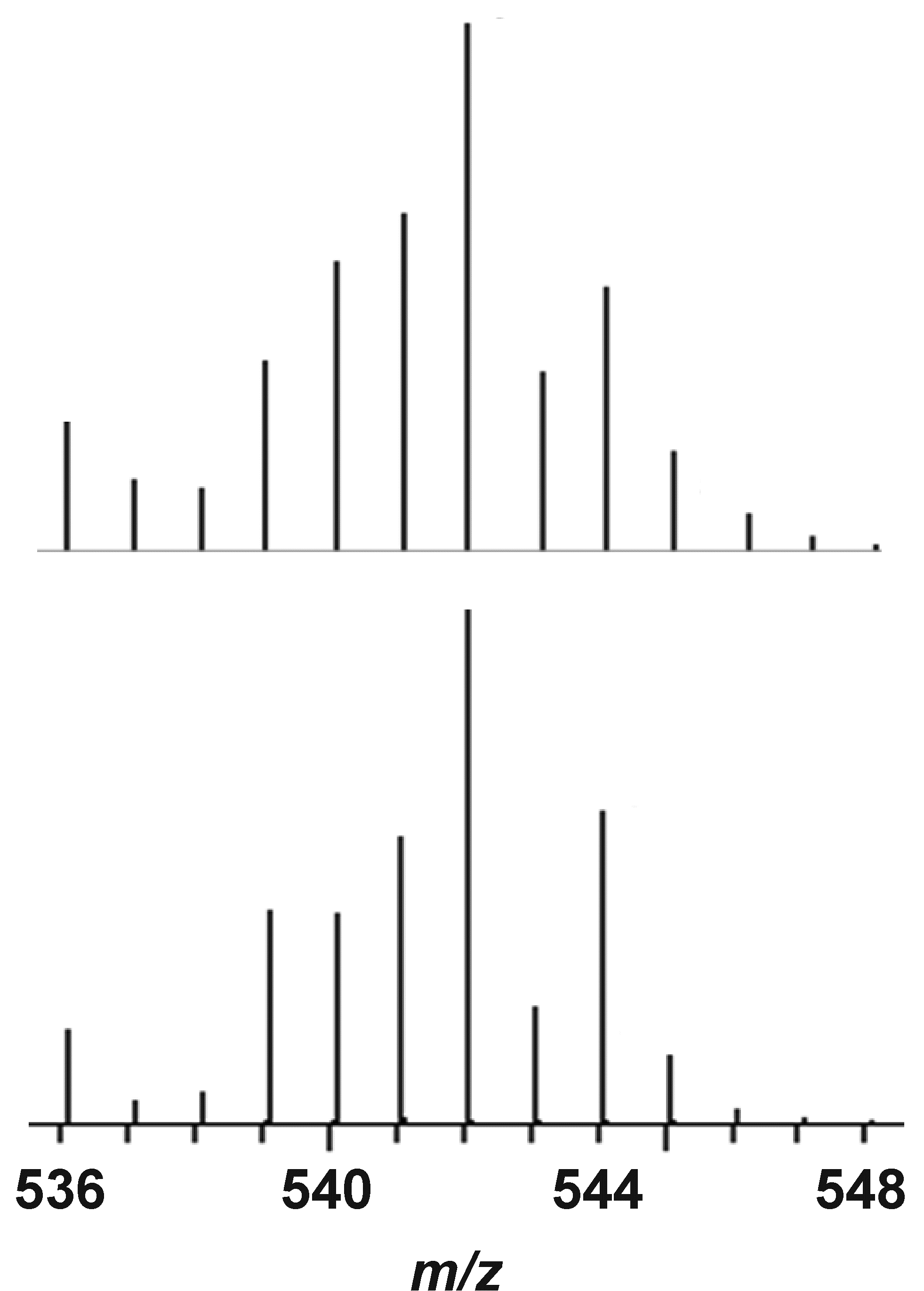

Supplement: S7 Fig — The spectrum was acquired on the 10% MeOH/H2O solution of 2 mixed with one molar equivalent of GSH. (TIF) [file pone.0143871.s007.tif]

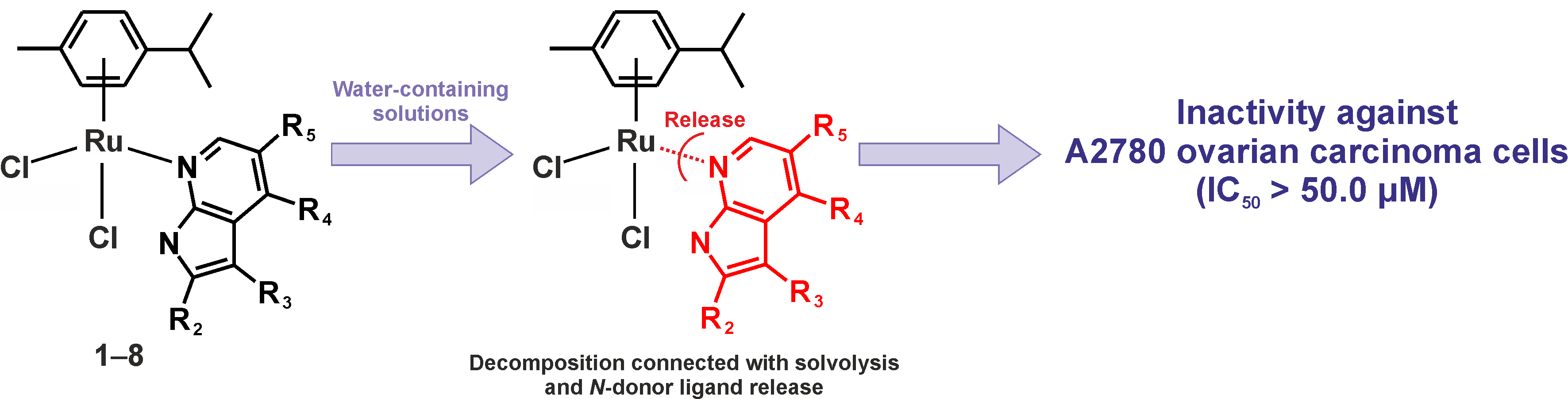

Supplement: S8 Fig — (TIF) [file pone.0143871.s008.tif]
