# Supplementary material for: Organometallic Half-Sandwich Dichloridoruthenium(II) Complexes with 7-Azaindoles: Synthesis, Characterization and Elucidation of Their Anticancer Inactivity against A2780 Cell Line
Source: PLoS One. 2015 Nov 25;10(11):e0143871. doi: 10.1371/journal.pone.0143871 (PMC4659567; doi:10.1371/journal.pone.0143871)
Supplement: S1 Table — (PDF) [file pone.0143871.s009.pdf]

Crystal data and structure refinement for [Ru(η^6-*p*-cym)(*2Me4Cl*aza)Cl_2] (**6**)

Empirical formula	$C_{18}H_{21}N_2Cl_3Ru$
Formula weight	472.79
Temperature (K)	120(2)
Wavelength (Å)	0.71073
Crystal system, space group	Monoclinic, *P2₁/n*
Unit cell dimensions	
a (Å)	12.5646(10)
b (Å)	7.7397(6)
c (Å)	20.017(2)
α (°)	90
β (°)	107.054(2)
γ (°)	90
V (Å^3)	1862.0(3)
Z, D_{calc} (g cm^{-3})	4, 1.687
Absorption coefficient (mm^{-1})	1.275
Crystal size (mm)	0.20 × 0.20 × 0.20
F (000)	952
θ range for data collection (°)	$2.13 \leq \theta \leq 26.00$
Index ranges (*h, k, l*)	$-15 \leq h \leq 15$
	$-9 \leq k \leq 8$
	$-24 \leq l \leq 24$
Reflections collected/unique	12698/3611 (R_{int} = 0.0286)
Data/restraints/parameters	3611/0/222
Goodness–of–fit on F^2	1.027
Final *R* indices [I>2σ(I)]	R_1 = 0.0215
	wR_2 = 0.0583
R indices (all data)	R_1 = 0.0228
	wR_2 = 0.0588
Largest peak and hole (e Å^{-3})	0.663, –0.543
