# Supplementary material for: Organometallic Half-Sandwich Dichloridoruthenium(II) Complexes with 7-Azaindoles: Synthesis, Characterization and Elucidation of Their Anticancer Inactivity against A2780 Cell Line
Source: PLoS One. 2015 Nov 25;10(11):e0143871. doi: 10.1371/journal.pone.0143871 (PMC4659567; doi:10.1371/journal.pone.0143871)
Supplement: S2 Table — (PDF) [file pone.0143871.s010.pdf]

Selected bond lengths (Å) and angles (°) of non-covalent contacts detected in the crystal structure of [Ru(η^6-*p*-cym)(*2Me4Cl*aza)Cl_2] (**6**)

Contact	*d*(D···A) (Å)	<(D–H···A) (°)
C3–H3···Cl2[i]	3.636	132.31(14)
C12–H12···C6[ii]	3.486(3)	125.64(12)
C13–H13···C19[ii]	3.664(3)	145.76(12)
C13···Cl1[iii]	3.397(2)	
C16–H16···Cl2[iv]	3.566(2)	161.30(10)
C18–H18A···C5[ii]	3.831(3)	160.28(14)°
C20–H20A···C16[v]	3.545(3)	138.43(13)°

Symmetry codes: i) 0.5-x, y-0.5, 0.5-z; ii) 1.5-x, y-0.5, 0.5-z); iii) x, y-1, z; iv) 1-x, 1-y, -z; v) 1-x, -y, -z
