# Supplementary material for: Organometallic Half-Sandwich Dichloridoruthenium(II) Complexes with 7-Azaindoles: Synthesis, Characterization and Elucidation of Their Anticancer Inactivity against A2780 Cell Line
Source: PLoS One. 2015 Nov 25;10(11):e0143871. doi: 10.1371/journal.pone.0143871 (PMC4659567; doi:10.1371/journal.pone.0143871)
Supplement: S1 Text — (PDF) [file pone.0143871.s011.pdf]

The results of FTIR spectroscopy of the studied complexes 1–8

[Ru(η^6-*p*-cym)(aza)Cl_2] (**1**): IR (ν_{ATR}/cm^{-1}):3273s, 3085m, 3055m, 2968m1589m, 1505w, 1466w, 1431m, 1410m, 1372m, 1341m, 1315m, 1272s, 1203w, 1090w, 1055m, 1036w, 1003w, 883w, 853w, 799s, 740s, 635m, 564m. ESI+ MS (methanol, *m/z*): 576.9 (calc. 576.9; 100%; [Ru_2(η^6-*p*-cym)$_2Cl_3$]$^+$), 570.9 (calc. 571.0; 100%; [Ru_2(η^6-*p*-cym)$_2Cl_2$(OCH_3)]$^+$), 542.9 (calc. 543.0; 70%; {[Ru_2^{1+}(η^6-*p*-cym)$_2Cl_2$]+H}$^+$), 507.0 (calc. 507.0; 50%; [Ru_2^{1+}(η^6-*p*-cym)$_2$Cl]$^+$), 388.9 (calc. 389.0; 20%; {[Ru(η^6-*p*-cym)(aza)Cl]}$^+$), 353.1 (calc. 353.0; 30%; {[Ru(η^6-*p*-cym)(aza)]–H}$^+$), 271.0 (calc. 271.0; 70%; {[Ru(η^6-*p*-cym)Cl]}$^+$), 119.2 (calc. 119.1; 15%; {(aza)+H}$^+$).

[Ru(η^6-*p*-cym)(*3Cl*aza)Cl_2] (**2**): IR (ν_{ATR}/cm^{-1}):3270s, 3140s, 3050w, 2963m, 2925m, 2868m, 1583s, 1488m, 1472m, 1434s, 1337m, 1312s, 1196m, 1082s, 1054m, 1029w, 1007s, 868w, 794m, 757m, 669w, 639w. ESI+ MS (methanol, *m/z*): 576.9 (calc. 576.9; 100%; [Ru_2(η^6-*p*-cym)$_2Cl_3$]$^+$), 570.9 (calc. 571.0; 100%; [Ru_2(η^6-*p*-cym)$_2Cl_2$(OCH_3)]$^+$), 542.9 (calc. 543.0; 90%; {[Ru_2^{1+}(η^6-*p*-cym)$_2Cl_2$]+H}$^+$), 507.0 (calc. 507.0; 70%; [Ru_2^{1+}(η^6-*p*-cym)$_2$Cl]$^+$), 422.9 (calc. 423.0; 20%; {[Ru(η^6-*p*-cym)(*3Cl*aza)Cl]}$^+$), 387.0 (calc. 387.0; 20%; {[Ru(η^6-*p*-cym)(*3Cl*aza)]–H}$^+$), 271.0 (calc. 271.0; 95%; {[Ru(η^6-*p*-cym)Cl]}$^+$), 153.1 (calc. 153.0; 20%; {(*3Cl*aza)+H}$^+$).

[Ru(η^6-*p*-cym)(*3I*aza)Cl_2] (**3**): IR (ν_{ATR}/cm^{-1}):3284s, 3123m, 3051m, 1958s, 2867m, 1708w, 1582s, 1487m, 1427s, 1324m, 1310m, 1272m, 1227w, 1184w, 1083s, 1054w, 1028w, 970s, 868m, 796m, 756m, 659m, 629w, 597w. ESI+ MS (methanol, *m/z*): 576.9 (calc. 576.9; 100%; [Ru_2(η^6-*p*-cym)$_2Cl_3$]$^+$), 570.9 (calc. 571.0; 100%; [Ru_2(η^6-*p*-cym)$_2Cl_2$(OCH_3)]$^+$), 542.9 (calc. 543.0; 95%; {[Ru_2^{1+}(η^6-*p*-cym)$_2Cl_2$]+H}$^+$), 507.0 (calc. 507.0; 50%; [Ru_2^{1+}(η^6-*p*-cym)$_2$Cl]$^+$), 514.8 (calc.

514.9; 50%; {[Ru(η^6-*p*-cym)(*3I*aza)Cl]}$^+$: [Ru(η^6-*p*-cym)(*3I*aza)(OCH_3)]$^+$ (2:1)), 271.0 (calc. 271.0; 70%; {[Ru(η^6-*p*-cym)Cl]}$^+$), 245.0 (calc. 244.9; 20%; {(*3I*aza)+H}$^+$).

[Ru(η^6-*p*-cym)(*5Br*aza)Cl_2](**4**): IR (ν_{ATR}/cm^{-1}):3251s, 3100m, 3035w, 2968w, 2872w, 1578m, 1504m, 1464s, 1416m, 1378m, 1288s, 1270m, 1190w, 1070w, 1057w, 1032w, 931m, 886w, 855m, 786w, 744m, 690w, 629w, 588w. ESI+ MS (methanol, *m/z*): 576.9 (calc. 576.9; 95%; [Ru_2(η^6-*p*-cym)$_2Cl_3$]$^+$), 570.9 (calc. 571.0; 95%; [Ru_2(η^6-*p*-cym)$_2Cl_2$(OCH_3)]$^+$), 542.9 (calc. 543.0; 100%; {[Ru_2^{1+}(η^6-*p*-cym)$_2Cl_2$]+H}$^+$), 507.0 (calc. 507.0; 70%; [Ru_2^{1+}(η^6-*p*-cym)$_2$Cl]$^+$), 466.9 (calc. 466.9; 30%; {[Ru(η^6-*p*-cym)(*5Br*aza)Cl]}$^+$), 271.0 (calc. 271.0; 80%; {[Ru(η^6-*p*-cym)Cl]}$^+$), 197.1 (calc. 197.0; 5%; {(*5Br*aza)+H}$^+$).

[Ru(η^6-*p*-cym)(*5F*aza)Cl_2](**5**): IR (ν_{ATR}/cm^{-1}):3313s, 3087m, 3044m, 2975m, 2918m, 2873m, 1595m, 1498s, 1468s, 1425m, 1380m, 1337s, 1285s, 1241m, 1206m, 1137m, 1088w, 1059w, 1037w, 988m, 933m, 882m, 856m, 803m, 779m, 730s, 689w, 632w. ESI+ MS (methanol, *m/z*): 576.9 (calc. 576.9; 100%; [Ru_2(η^6-*p*-cym)$_2Cl_3$]$^+$), 570.9 (calc. 571.0; 100%; [Ru_2(η^6-*p*-cym)$_2Cl_2$(OCH_3)]$^+$), 542.9 (calc. 543.0; 95%; {[Ru_2^{1+}(η^6-*p*-cym)$_2Cl_2$]+H}$^+$), 507.0 (calc. 507.0; 25%; [Ru_2^{1+}(η^6-*p*-cym)$_2$Cl]$^+$), 406.9 (calc. 407.0; 10%; [Ru(η^6-*p*-cym)(*5F*aza)Cl]}$^+$), 271.0 (calc. 271.0; 65%; {[Ru(η^6-*p*-cym)Cl]}$^+$), 137.1 (calc. 137.2; 10%; {(*5F*aza)+H}$^+$).

[Ru(η^6-*p*-cym)(*2Me4Cl*aza)Cl_2] (**6**): IR (ν_{ATR}/cm^{-1}):3168s, 3051m, 2966m, 2927m, 2871w, 1581m, 1546m, 1466w, 1429m, 1399w, 1381w, 1336m, 1305m, 1248m, 1194m, 1148w, 1057w, 964m, 883m, 793m, 755w, 722w, 672w, 637w, 618w, 665w. ESI+ MS (methanol, *m/z*): 576.9 (calc. 576.9; 100%; [Ru_2(η^6-*p*-cym)$_2Cl_3$]$^+$), 570.9 (calc. 571.0; 100%; [Ru_2(η^6-*p*-cym)$_2Cl_2$(OCH_3)]$^+$), 542.9 (calc.

543.0; 95%; {[Ru_2^{1+}(η^6-*p*-cym)$_2$$Cl_2$]+H}$^+$), 507.0 (calc. 507.0; 45%; [Ru_2^{1+}(η^6-*p*-cym)$_2$Cl]$^+$), 436.9 (calc. 437.0; 20%; {[Ru(η^6-*p*-cym)(*2Me4Cl*aza)Cl]}$^+$), 401.1 (calc. 401.0; 10%; {[Ru(η^6-*p*-cym)(*2Me4Cl*aza)]–H}$^+$), 271.0 (calc. 271.0; 75%; {[Ru(η^6-*p*-cym)Cl]}$^+$), 167.1 (calc. 167.0; 20%; {(*2Me4Cl*aza)+H}$^+$).

[Ru(η^6-*p*-cym)(*3Cl5Br*aza)Cl_2] (**7**): IR (ν_{ATR}/cm^{-1}):3190s, 3142s, 3096m, 3026m, 2969s, 2871m, 1614m, 1596m, 1577m, 1505w, 1463s, 1417m, 1402m, 376m, 1324m, 1274s, 1228w, 1197m, 1116w, 1089w, 1059w, 1018s, 904m, 880m, 855s, 807m, 792w, 759w, 715m, 633w, 594m. ESI+ MS (methanol, *m/z*): 576.9 (calc. 576.9; 100%; [Ru_2(η^6-*p*-cym)$_2$$Cl_3$]$^+$), 570.9 (calc. 571.0; 70%; [Ru_2(η^6-*p*-cym)$_2$$Cl_2$($OCH_3$)]$^+$), 542.9 (calc. 543.0; 70%; {[Ru_2^{1+}(η^6-*p*-cym)$_2$$Cl_2$]+H}$^+$), 507.0 (calc. 507.0; 45%; [Ru_2^{1+}(η^6-*p*-cym)$_2$Cl]$^+$), 467.0 (calc. 467.1; 10%; {[Ru(η^6-*p*-cym)(*3Cl5Br*aza)]–H}$^+$), 271.0 (calc. 271.0; 45%; {[Ru(η^6-*p*-cym)Cl]}$^+$), 232.9 (calc. 233.0; 10%; {(*3Cl5Br*aza)+H}$^+$).

[Ru(η^6-*p*-cym)(*3I5Br*aza)Cl_2] (**8**): IR (ν_{ATR}/cm^{-1}): 3184s, 301s, 2965s, 2927m, 2871m, 1626w, 1573m, 1502w, 1458s, 1407m, 1375m, 1321m, 1271s, 1225w, 1199w, 1156w, 1113w, 1089w, 1053w, 984m, 905w, 873m, 813w, 787w, 759w, 628w, 592m. ESI+ MS (methanol, *m/z*): 628.9 (calc. 628.8; 5%; {[Ru(η^6-*p*-cym)(*3I5Br*aza)Cl_2]+H}$^+$), 592.8 (calc. 592.8; 15%, [Ru(η^6-*p*-cym)(*3I5Br*aza)Cl]$^+$), 576.9 (calc. 576.9; 70%; [Ru_2(η^6-*p*-cym)$_2$$Cl_3$]$^+$), 570.9 (calc. 571.0; 70%; [Ru_2(η^6-*p*-cym)$_2$$Cl_2$($OCH_3$)]$^+$), 542.9 (calc. 543.0; 100%; {[Ru_2^{1+}(η^6-*p*-cym)$_2$$Cl_2$]+H}$^+$), 507.0 (calc. 507.0; 50%; [Ru_2^{1+}(η^6-*p*-cym)$_2$Cl]$^+$), 324.9 {(calc. 324.9; 15%; {(*3I5Br*aza)+H}$^+$), 271.0 (calc. 271.0; 50%; {[Ru(η^6-*p*-cym)Cl]}$^+$).
